# Dynamic switching of cell–substrate contact sites allows gliding diatoms to modulate the curvature of their paths

**DOI:** 10.1073/pnas.2506122123

**Published:** 2026-04-01

**Authors:** Stefan Golfier, Veikko F. Geyer, Leon Lettermann, Ulrich S. Schwarz, Nicole Poulsen, Stefan Diez

**Affiliations:** ^a^B CUBE - Center for Molecular Bioengineering, TUD Dresden University of Technology, Dresden 01307, Germany; ^b^Cluster of Excellence Physics of Life, TUD Dresden University of Technology, Dresden 01062, Germany; ^c^Institute for Theoretical Physics, Heidelberg University, Heidelberg 69120, Germany; ^d^Bioquant-Center, Heidelberg University, Heidelberg 69120, Germany; ^e^Max Planck Institute of Molecular Cell Biology and Genetics, Dresden 01307, Germany

**Keywords:** diatom gliding, cell motility, quantitative microscopy, mathematical modelling

## Abstract

The directed motility of unicellular organisms is critical for their survival and ecological success, yet the mechanisms that enable rigid-walled diatoms to dynamically reorient and alter the shape of their trajectories remain poorly understood. Here, we investigate the gliding motility of *Craspedostauros australis*, a raphid pennate diatom that moves rapidly across submerged surfaces using an intracellular actomyosin motility complex and the secretion of adhesive extracellular polymeric substances (EPS) strands through slit-like openings termed raphes. Using high-precision single-cell tracking, scanning electron microscopy (SEM), interference reflection microscopy (IRM), and mathematical modeling, we reveal how diatoms achieve diverse path curvatures by dynamically modulating the location of raphe–substrate contact and switching between one- and two-raphe branch contact gliding. Our results indicate that local curvature variations along the raphes dictate trajectory shapes, with one-raphe branch contact gliding producing highly curved paths, while two-raphe branch contact gliding results in paths of lower curvature. IRM imaging further confirms that transitions between these gliding modes underlie abrupt changes in path curvature and cell reorientation. This dynamic raphe-switching mechanism is conserved across cell sizes and correctly predicts the increased path curvatures observed in smaller cells according to their more pronounced local raphe curvature. By quantitatively linking raphe geometry, cell–substrate attachment dynamics and motility patterns, our study provides insights into the motility mechanism that allows diatoms to adapt their movement to complex environments.

## Significance

Diatoms are photosynthetic microalgae and major primary producers across diverse aquatic habitats. Many diatom species navigate their complex environments using gliding motility, facilitated by slit-like openings (raphes) in their rigid silica cell wall. Unlike many motile cells, diatoms achieve directional flexibility without cell deformation or external appendages, yet the underlying mechanism remains unknown. Here, we tracked the motility of single diatom cells and correlated the curvature of their trajectories with the local geometry of their raphes. We show that diatoms dynamically modulate their cell–substrate contact points along the raphe, allowing them to control the curvature of their trajectories. This mechanism provides a fundamental explanation for diverse motility patterns observed across diatom species and resolves a long-standing question in diatom locomotion.

Motility is a fundamental hallmark of life across all scales. From single cells to large organisms, diverse mechanisms have evolved to traverse space in search of food, mating partners, and favorable conditions while evading predation and hazardous environments. Free-swimming single-celled organisms often rely on beating cilia and flagella (as in some green algae or sperm cells) or rotating flagella (as in some bacteria) that allow for speeds of several 100 µm/s (1). In contrast, surface-bound motility such as amoeboid crawling, apicomplexan gliding, or bacterial twitching is slower (2–4). An intriguing exception are diatoms, an abundant class of unicellular microalgae encased in a rigid silica cell wall, many of which exhibit rapid gliding motility of up to 35 µm/s along complex trajectories on submerged surfaces (5). Remarkably, they achieve this without extracellular appendages or cell deformation (6, 7).

**Fig. 1.**
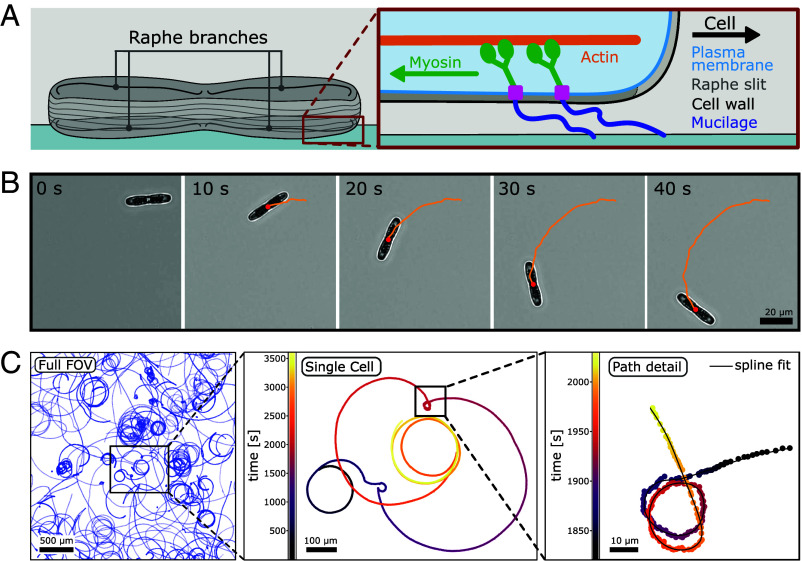
Single-cell tracking of motile *Craspedostauros australis*. (*A*) Schematic representation of a diatom gliding on a glass surface. *Inset*: Current understanding of the diatom adhesion motility complex (AMC). Intracellular myosin motors (green) couple via a transmembrane complex (pink) to adhesive EPS strands (dark blue), which are secreted through the raphe in the cell wall (gray) and adhere to the glass surface. Cell motility is achieved through myosin movement on intracellular actin cables (orange), leading to tension on the adhesive EPS strands that propels the cell forward. (*B*) Selected timelapse images (acquired at 3 fps) of the motility of an individual diatom cell over 40 s. The trajectory (orange) is derived from high-precision tracking of the cell center of mass (red) in each image. (*C*) Trajectories of multiple *C. australis* cells over one hour (acquired at 1 fps). *Left* panel: Full field of view, displaying over 100 individual single-cell trajectories. Mid panel: Typical trajectory of an individual cell displaying several changes in path curvature, color-coded for time. *Right* panel: Zoom-in into the same track illustrating the spatial and temporal resolution employed in this study as well as a spline fit used to quantify path curvature.

Diatoms are broadly classified into two major groups based on cell shape: radially symmetric “centric” diatoms, which exist mainly as free-floating phytoplankton, and bilaterally symmetric “pennate” diatoms, which are predominantly benthic, colonizing sunlit underwater surfaces. A subgroup of pennate diatom species is highly motile, which is enabled by a system of longitudinal slits in their cell wall, termed “raphe.” These raphid pennate diatoms adhere to surfaces via the secretion of adhesive extracellular polymeric substances (EPS) strands through the raphe (8–12). The forces for motility are likely produced by an intracellular actomyosin force-generating motor translocating the substrate-bound adhesive EPS strands along the raphe in order to propel the cell forward, termed the adhesion motility complex (AMC) (Fig. 1*A*, for review see (6, 13)). Despite numerous theories, the exact mechanism of diatom propulsion has yet to be resolved.

The ecological success of raphid pennate diatoms in their complex and dynamic environments can in large parts be attributed to their active motility, enabling them to colonize new aquatic habitats, optimize light exposure (14–20), enhance nutrient uptake (21–23), and pursue reproductive strategies (24). The pronounced photo- and chemotactic motility of diatoms (25, 26) is based on their ability to navigate along strikingly complex paths, featuring dynamic changes in path curvature and directional reversals, allowing them to quickly reorient their movement in response to external cues (5, 27, 28). However, how cells that are encased in a rigid cell wall achieve dynamic directional flexibility without cell deformation and extracellular appendages remains poorly understood.

Several studies have proposed that the shape of a diatom’s trajectory is governed by the geometry of its raphe system (28–33). Specifically, it has been suggested that traction is exerted over only a few micrometers of the raphe system in contact with the substrate at any given time and that the local curvature of this active zone dictates the cell’s trajectory at that moment (33). By shifting this active zone along the raphe and thereby engaging a raphe segment of different curvature, diatoms may be able to dynamically adjust their path curvature, enabling directional flexibility despite their rigid cell wall. Nevertheless, a mechanistic understanding of how local raphe geometry determines trajectory shape and how cells transition between different path curvatures is lacking.

Here, we introduce a refined model of diatom gliding motility that quantitatively links raphe geometry, cell–substrate attachment dynamics and cell size to the path curvature of *Craspedostauros australis*, a model species for studying diatom adhesion and motility (34, 35). This diatom species features two external raphe branches per valve (separated by a central nodule). Using high-precision single-cell tracking over long timeframes, scanning electron microscopy (SEM) of cell wall geometries, and interference reflection microscopy (IRM) of cell–substrate contact sites along with a mathematical model and computer simulations, we find that the observed spectrum of path curvatures can be explained by distinct modes of gliding motility, each enabling traction in different regions of the raphe. Direct correlations between cell–substrate contact sites and path curvature confirm that diatoms dynamically transition between these modes, leading to abrupt changes in path curvature and cell reorientation. Based on these findings, we propose and mathematically validate a model of diatom motility that accounts for the observed cell-size dependency of *C. australis* trajectories and advances our fundamental understanding of cellular movement.

## Results

### Single-Cell Tracking of Motile Diatoms Reveals Dynamic Changes in Path Curvature.

To investigate the motility characteristics of *C. australis*, we settled cells grown in artificial seawater medium onto hydrophobically coated glass coverslips and recorded time-lapse movies using brightfield microscopy (Fig. 1*B* and *Materials and Methods*). Each field of view (3.2 mm × 3.2 mm) contained up to 100 cells, which were imaged for time periods of up to one hour with a frame rate of 1 frame-per-second (fps) (Movie S1). For every frame, we extracted the spatial coordinates of each cell by determining the position of its center of mass, allowing us to reconstruct trajectories of individual cells (Fig. 1*C*). Over short timescales (tens of seconds to minutes), the trajectories of single cells typically followed circular paths with conserved radii, as was previously observed in other raphid diatoms (6, 36). However, over longer timescales, individual cell trajectories exhibited a remarkable variability in their path curvatures (Movie S2). We frequently observed abrupt and substantial changes in path curvature, both from quasi-straight to highly curved paths and vice versa. Additionally, we noticed a strong variability in the overall shapes of the trajectories between different cells (*SI Appendix*, Fig. S1), which persisted across different surface chemistries (*SI Appendix*, Fig. S2). This is surprising, because we studied cells derived from monoclonal cell cultures (i.e. with similar raphe morphology) and performed experiments under isotropic conditions (i.e. under homogeneous illumination, homogeneous treatment of the substrate surface and in the absence of chemical gradients). In light of previous literature suggesting raphe geometry as the basis of path shape (29,28, 32, 33), we were wondering about the source of heterogeneity in path shapes from a cell population that should feature similar raphe geometries. We thus sought to identify potential correlations between the motility parameters that would allow us to formulate testable hypotheses on the underlying mechanisms.

### Path Curvature Is Correlated With Cell Velocity and Orientation.

**Fig. 2.**
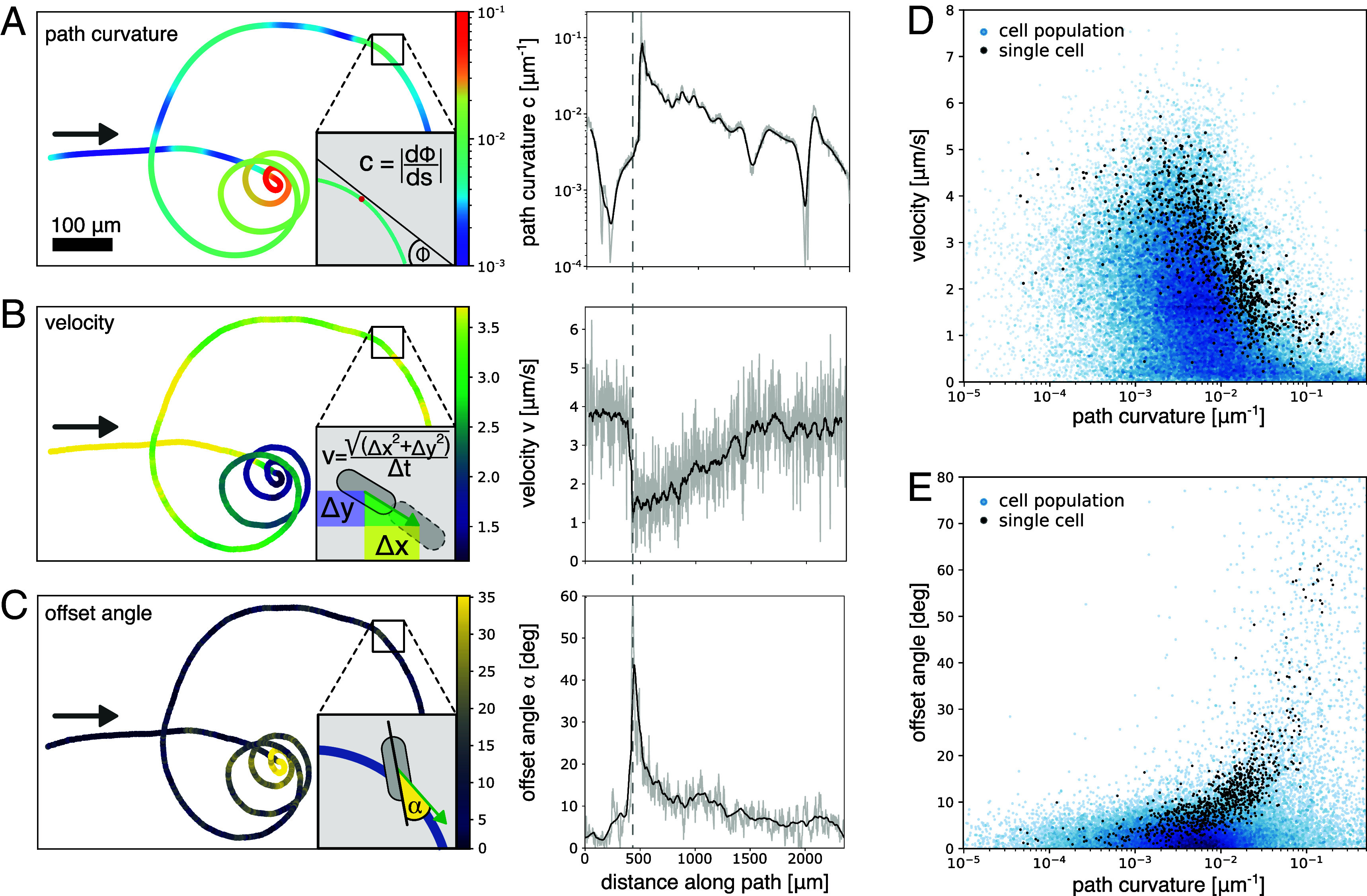
Parameters to quantify the motility of *C. australis* cells. (*A*) Quantification of path curvature for a single cell trajectory. The gray arrow indicates the direction of travel. Inset depicts the definition of path curvature (*Materials and Methods*). Color-code indicates path curvature. (*Right*) Path curvature along the path with raw data in light gray, rolling average with window size of 4 µm in black. (*B*) Quantification of cell velocity v for the same track as in *A*, color-coded for cell velocity. Inset depicts the definition of velocity along the path with the velocity vector in green (*Materials and Methods*). (*Right*) Cell velocity along the path with raw data in light gray and rolling average with window size of 4 µm in black. (*C*) Quantification of offset angle α for the same track as in *A*, color-coded for offset angle. Inset depicts the definition of the offset angle (yellow) as the absolute difference between the orientation of the cells’ long axis (black line) and the direction of travel (green arrow) (*Materials and Methods*). (*Right*) Offset angle along the path with raw data in light gray and rolling average with window size of 4 µm in black. The dashed gray line denotes the distance along the path where a strong reduction in velocity and an increase in offset angle occur simultaneously, followed by the onset of an increased path curvature. (*D*) Cell velocity over path curvature for the single cell track from *A*–*C* (black dots) and a cell population of 196 cells (blue dots, logarithmic density colormap) over timescales of up to one hour. (*E*) Cell offset angle over path curvature for the same single cell track (black dots) and the same cell population (blue dots, logarithmic density colormap).

To identify potential correlations between the motility parameters, we quantitatively analyzed the path curvature and the velocity of each individual cell along its trajectory, as well as the cell’s orientation with respect to its direction of travel (Fig. 2 *A*–*C* and *Materials and Methods* for the respective definitions). For simplicity, we first discuss our results for an exemplary trajectory of a single cell tracked over 890 s (approximately 15 min), during which the cell traversed a total distance of 2,400 µm (Movie S3). Initially the cell followed a trajectory with low curvature (curvature of about 0.001 µm^−1^). After 450 µm along the path, the path curvature suddenly increased by more than two orders of magnitude (Fig. 2*A*, right panel vertical dashed gray line). Over the next 2,000 µm, the path curvature gradually decreased, resulting in a spiral-shaped trajectory with increasing radius. The velocity profile of this trajectory reveals that the cell significantly slowed down before transitioning from a path with low curvature (quasi-straight) to a path with high curvature (tight circles) (Fig. 2*B*). Afterward, it gradually accelerated as the path curvature decreased, regaining its initial velocity once the path curvature reached the initial level. Furthermore, we found that along path segments of high curvature, the orientation of the cell’s long axis displayed a significant offset (up to 60 degrees) with respect to the direction of travel (Fig. 2*C* and Movie S4). Closer inspection revealed that just before the sudden increase in path curvature, the back of the cell started to spin around its leading apex, resulting in an abrupt increase in the cell’s offset angle. Both, the strong reduction in velocity and the increase in offset angle, occur simultaneously (see gray dashed line) and precede the onset of increased path curvature. This behavior was observed across multiple cells when path curvature increased sharply, suggesting a dynamic mechanism that trades speed for directional flexibility (Movie S5).

When quantitatively relating the measured motility parameters to each other, we found that cell velocity was anticorrelated to path curvature (Spearman’s rank correlation coefficient of −0.73), and decreased exponentially with increasing path curvature (Fig. 2*D*, black dots and *SI Appendix*, Fig. S3*A*). Consequently, the cell displayed maximum velocities (up to 8 µm/s) along quasi-straight path segments (curvature < 0.001 µm^−1^) and slowed down significantly once the curvature increased above 0.03 µm^−1^ (about 1/cell length). In contrast, the cells’ offset angle was correlated with path curvature (Spearman’s rank correlation coefficient of 0.85), and increased exponentially with increasing path curvature, exhibiting large offset angles along highly curved path segments and small offset angles along quasi-straight path segments (Fig. 2*E*, black dots and *SI Appendix*, Fig. S3*B*). Conversely, cell velocity decreased with increasing offset angle (*SI Appendix*, Fig. S3*C*). These relationships between path curvature, cell velocity, and offset angle, are, albeit with higher variability, also observed on a population level (Fig. 2 *D* and *E* blue dots, 196 cells tracked over timescales of up to one hour, showing a characteristic velocity peak around 2 µm/s, *SI Appendix*, Fig. S3*D*). The pronounced variability in the population data, relative to the single-cell data presented, arises primarily from differences between cells rather than from a lack of correlation within individual cells (*SI Appendix*, Fig. S4). Our datasets also exhibit directional reversals, an additional intriguing feature that occurs approximately once every 1 mm along a *C. australis* trajectory on hydrophobic coverslips – likely reflecting tug-of-war dynamics of the intracellular actomyosin motility complexes (37). Because path curvature is undefined at these reversal points, they were excluded from our trajectory analysis. Taken together, our data indicate that it is unlikely for a cell to display rapid movement along stretches of high path curvature and similarly unlikely for a cell to show high offset angles while moving along path sections of low curvature.

Our observations show that the path curvature of motile *C. australis* cells is highly variable and strongly correlates with cell velocity and offset angle. In particular, the high offset angles observed in path segments with high curvature suggest that in this situation, the cell pivots around a short, discrete segment of the raphe. In this case, force transduction required for processive movement may be impeded, which likely explains the observed low velocities. On the other hand, the low offset angles observed along segments with low path curvature suggest that longer sections of the raphe engage with the substrate, enabling greater forces for motility and better alignment between actomyosin machinery and EPS strands, and hence higher velocities. As such, our findings support the hypothesis that the motility of diatom cells might be governed by short segments of the raphe system in contact with the substrate (33). However, the highly dynamic nature of the observed trajectories contrasts with the structural rigidity of the raphe system, underscoring the need for a dynamic mechanism that translates static local raphe geometry into adaptable path curvatures.

### Path Curvature Can Be Predicted From Raphe Geometry by Distinct Modes of Gliding Motility.

**Fig. 3.**
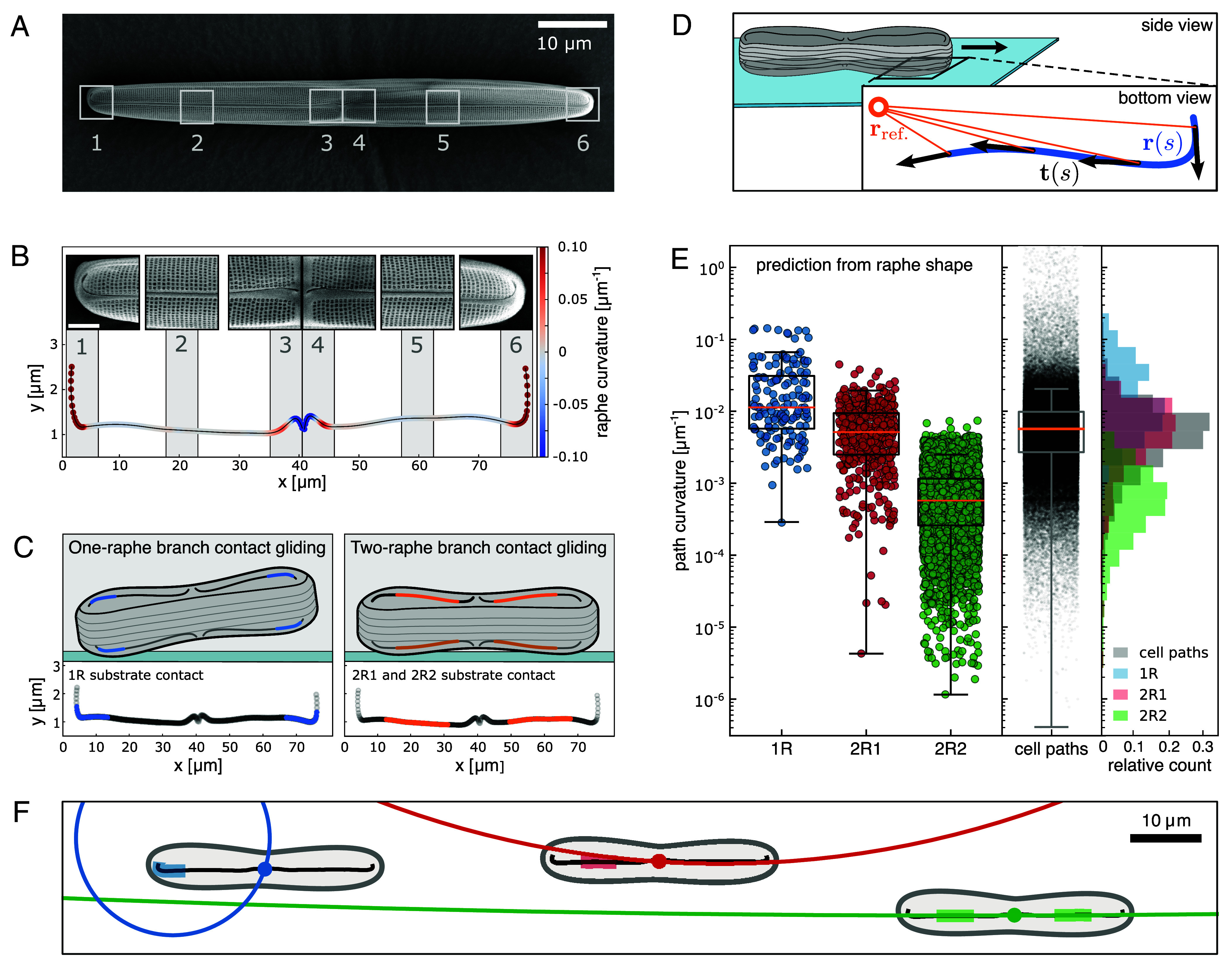
Predicting *C. australis* path curvature. (*A*) Scanning electron microscopy (SEM) image of the silica cell wall of one *C. australis* cell. (*B*) Quantification of raphe curvature along the two raphe branches segmented from the cell wall in *A*, with regions of interest (1–6) to illustrate the local appearance of the raphe. (*C*) Modes of diatom gliding motility. (*Left*) One-raphe branch contact gliding (1R) requires the cell to pivot up (away from the substrate) in order to allow only a single stretch of one raphe branch to contact the substrate. Cell wall geometry restricts the regions of contact with the substrate to about the terminal third of each raphe branch, illustrated in blue. The terminal 1 µm in each raphe branch was excluded as it is filled with silica. (*Right*) During two-raphe branch contact gliding (2R) the cell makes simultaneous contact to the substrate with two segments along the raphe branches in both halves of the cell. Cell wall geometry restricts the regions of substrate contact to the central half of each raphe branch, highlighted in orange. Forces for gliding motility may be produced along only one segment in one of the two raphe branches (2R1) or along two segments in both raphe branches simultaneously (2R2). The central nodule and its vicinity cannot make contact to the substrate as the cell wall curves inward in these regions and was thus excluded from analysis. (*D*) Schematics of the mathematical model of diatom gliding. An active segment of the raphe (blue) is parameterized as $r\left(s\right)$ relative to a reference position $r_{\mathrm{ref}}$, the center of the cell, where s is arc length. We assume constant traction with the substrate along the tangent vector r(s) of the active raphe segment, with frictional force proportional to the relative velocity between cell and substrate. (*E*) (*Left*) Spectra of path curvatures predicted by the mathematical model in *D*, considering 4 µm rolling windows as active segments within the one-raphe branch (blue dots) and two-raphe branch (red dots) contact zones, representing the predicted path curvatures for 1R and 2R1 gliding respectively. Predicted path curvatures for two-raphe branch contact gliding with simultaneous activity on both raphe branches (2R2) were obtained considering all combinations of 4 µm windows in the front and hind 2R raphe segments in our mathematical model (green dots). Significance testing returned significant differences (Mann–Whitney *U* test, *P* < 0.001) between the 1R and 2R1, the 1R and 2R2, as well as the 2R1 and 2R2 distributions, respectively. The raphe geometry analyzed in the mathematical model was obtained using SEM images of 18 diatom cell walls, prepared from the cell population in Fig. 2 *D* and *E*. Also shown is the spectrum of experimentally observed path curvatures from 196 trajectories from the cell population in Fig. 2 *D* and *E*, averaged over 4 µm windows along the path (*Middle*, black dots). (*Right*) Overlay of normalized histograms of predicted and observed path curvatures. (*F*) Mathematically predicted trajectories of the cell center for a small diatom cell, assuming traction along a 4 µm raphe segment in the 1R, 2R1, and 2R2 regions (blue, red, and green shaded areas), resulting in path curvatures of 0.065 µm^−1^ for 1R (radius of 15 µm, *Left* blue line), 0.0065 µm^−1^ for 2R1 (radius of 153 µm, *Middle* red line) and 0.00026 µm^−1^ for 2R2 gliding (radius of 3,714 µm, *Right* green line).

In diatom gliding motility, a myosin-mediated surface flow of substrate-bound adhesion molecules (EPS strands) along the raphe is converted into motion of the rigid cell body. We consequently hypothesize that the local shape of the raphe plays a central role in determining the curvature of the cellular trajectory. However, a physical framework that quantitatively links raphe geometry to path curvature has been lacking. To test whether local raphe shape alone can account for the observed path curvatures, we first segmented the raphe system of 20 cells from SEM images of the silica cell walls from cells of the population used to generate Fig. 2 *D* and *E*. Fig. 3*A* depicts an SEM image of a typical *C. australis* cell wall with the two raphe branches visible as dark ridges running along the long axis of the cell, interrupted by a silica bridge in the middle of the cell (central nodule). From the obtained *x-y*-data we quantified the local 2D raphe curvature (Fig. 3*B* and *SI Appendix*, Fig. S5*A* and *Materials and Methods*) and found that the local curvature along the raphe branches of each cell varies over several orders of magnitude and in clockwise and anticlockwise direction. We observed highly curved regions at the cells’ ends (terminal raphe fissures) and centers (close to the central nodule) as well as stretches of low curvature in between (Fig. 3*B*). In all cases, the raphe shapes overall followed the bilateral symmetry of the *C. australis* cell wall, displaying similar curvature patterns in both halves of the cell.

We also found that the valve surfaces with which the cells establish substrate contact are highly nonplanar, and as such the entire raphe system can never simultaneously come into contact with the flat coverslip surface. As recent observations suggest that only those parts of the raphe that can make direct contact to the substrate below a distance of 1 µm, possibly mediated by the EPS strands, are able to generate the necessary traction-forces required for motility (28, 33, 37), we sought to consider which parts of the raphe could actually contribute to gliding motility. Based on our SEM images of the lateral *C. australis* cell wall shape (*SI Appendix*, Fig. S5*B*), we heuristically subdivided the raphe branches into distinct contact zones, which could independently drive motility. When only one raphe branch contacts the substrate—a configuration we term one-raphe branch contact gliding (1R)—the cell must pivot upward, restricting substrate contact to the terminal ~30% of each raphe branch; otherwise, other portions of the raphe system would approach the coverslip (Fig. 3 *C*, *Left* panel and *SI Appendix*, Fig. S5 *B* and *C* blue region, *Materials and Methods*). In contrast, when the cell lies horizontally, the central ~50% of both raphe branches can simultaneously engage the substrate, a configuration we refer to as two-raphe branch contact gliding (2R) (Fig. 3 *C*, *Right* panel and *SI Appendix*, Fig. S5 *B* and *C* red region and dots, *Materials and Methods*). In this 2R configuration, however, it is not immediately evident whether traction is generated along only one raphe branch (2R1) or along both raphe branches concurrently (2R2). Recent work using the same diatom species indicates that myosin motors can operate in the same direction in both raphe branches during smooth gliding, consistent with simultaneous force generation along the entire raphe system (37). Motivated by this, we expanded upon earlier models that assumed a single active raphe zone determines path curvature (28, 33, 38) and hypothesized that in the two-raphe branch contact configuration (2R2) both contact zones—one in each raphe branch—may contribute to motility and thus jointly influence path curvature.

To compute the resulting path curvatures a cell would be traveling along for all three gliding scenarios (1R, 2R1, and 2R2), we adapted a mathematical model that earlier was devised for apicomplexan gliding (39). While this model has been used mainly to analyze the effect of self-organized surface flow patterns for the resulting motility patterns, here the direction of the flow is fixed by the geometrical shape of the raphes. The segmented raphes from our SEM data are described by a line parameterized in 2D, that allows us to analyze the raphe geometry within 4 µm subsections, which we assume as the size of an active raphe segment (Fig. 3*D* and *Materials and Methods*). We make the simplifying assumption that every part of the raphe considered active creates the same flow velocity tangential to the raphe (possibly by producing a constant motion of the adhesive EPS strands), while all other parts of the cell are considered to be decoupled. In contrast to the case of the apicomplexa, we thus do not consider any feedback between the environment and the gliding machinery of the cell. In particular, we neglect the mechanical properties of the EPS strands and the intracellular dynamics of the actomyosin machinery. However, similar to the case of the apicomplexa, the resulting motion is not trivial, because global motion of the rigid cell results from the integration of the local flows along the raphe system. Assuming overdamped motion and requiring force balance, we can solve mathematically for the resulting motion of the cell, yielding the translational and angular velocities of the cell body, that allow us to predict the curvature of the resulting path shape based on the local raphe geometry (see *Materials and Methods* for details).

For 1R gliding, the mathematical model predicts that the highly curved raphe geometries along the 1R raphe segments translate into high path curvatures (1R median 0.012 µm^−1^) or turning radii of about one cell length (20 to 80 µm) (Fig. 3 *E*, *Left* panel 1R, blue dots). These small turning radii indeed correspond to the tightest curves we observed in cell trajectories, as depicted in Figs. 2*A* and 1*C*. In contrast, during 2R gliding with only one raphe branch providing forces for motility (2R1), our model predicts significantly lower path curvatures, with a median value of 0.005 µm^−1^ (Fig. 3 *E*, *Left* panel 2R1, red dots). This reduction in predicted path curvatures between 1R and 2R1 gliding highlights how the straighter raphe geometries in the 2R contact regions translate through our model into lower path curvatures. The predicted 2R1 turning radii of several cell lengths (hundreds of µm) correspond to the typical circular trajectories frequently observed in our cell tracking data. Finally, including equal contributions to motility in both 2R raphe segments into our model, 2R2 gliding yielded the lowest predicted path curvatures with a median of 0.0005 µm^−1^ (Fig. 3 *E*, *Left* panel 2R2, green dots). The resulting 2R2 turning radii of tens to hundreds of cell lengths correspond to the straightest paths in our trajectory datasets. However, the large spread in the 2R2 data indicates that even when both raphe branches simultaneously generate traction, the cell retains some directional flexibility, allowing for nuanced variations in path curvature.

To test our model predictions, we analyzed the actual path curvatures of the 196 diatom trajectories used to generate Fig. 2 *D* and *E* and obtained a distribution with a median curvature of 0.005 µm^−1^ (Fig. 3 *E*, *Center* panel, black dots). Comparing the predictions of our model to these experimentally obtained path curvatures, we found that the predicted path curvatures during one-raphe branch contact gliding (1R, blue dots) mapped only to the high-curvature path segments in our tracking dataset, yet failed to account for a large part of the observed path curvature spectrum below 0.005 µm^−1^. Conversely, the predicted path curvatures during two-raphe branch contact gliding (2R1 and 2R2, red and green dots) corresponded to the lower part of the measured path curvature spectrum, with 2R1 values matching closest the bulk of the cell path curvature distribution, but failed to cover the highly curved path segments frequently observed in the cells´ trajectories. We note that in order to quantitatively reproduce the distribution of the observed path curvatures from the predicted path curvatures, one would need to know the fractions of the trajectories where the cells move in 1R, 2R1, or 2R2. We do not have knowledge about this distribution and hence can only provide a qualitative agreement of predicted and observed path curvatures. We further noticed that a tiny fraction (~0.1%) of the observed path curvatures exceeds the maximum path curvatures predicted by our model. Inspecting the origin of these high path curvatures, we found that these result from sharp directional reversals in the cell trajectory data, which are not covered by the model. Despite this caveat, though, the predicted curvatures cover over 99.8% of the measured path curvature spectrum (Fig. 3 *E*, *Right* panel).

Taken together, our model suggests that only the combination of one-raphe branch and two-raphe branch contact gliding can account for the full range of path curvatures observed in our cell tracking data, suggesting that dynamic changes in path curvature originate from cells switching between these modes of gliding (Fig. 3*F*). This hypothesis is further supported by multimodal distributions of path curvature in the trajectories of single cells, suggesting switches between discrete modes of gliding (*SI Appendix*, Fig. S6). Consequently, we next sought a method to experimentally quantify cell–substrate attachment dynamics in concert with path curvature.

### Substantial Changes in Path Curvature Result From Switching Between One- and Two-Raphe Contact Gliding.

**Fig. 4.**
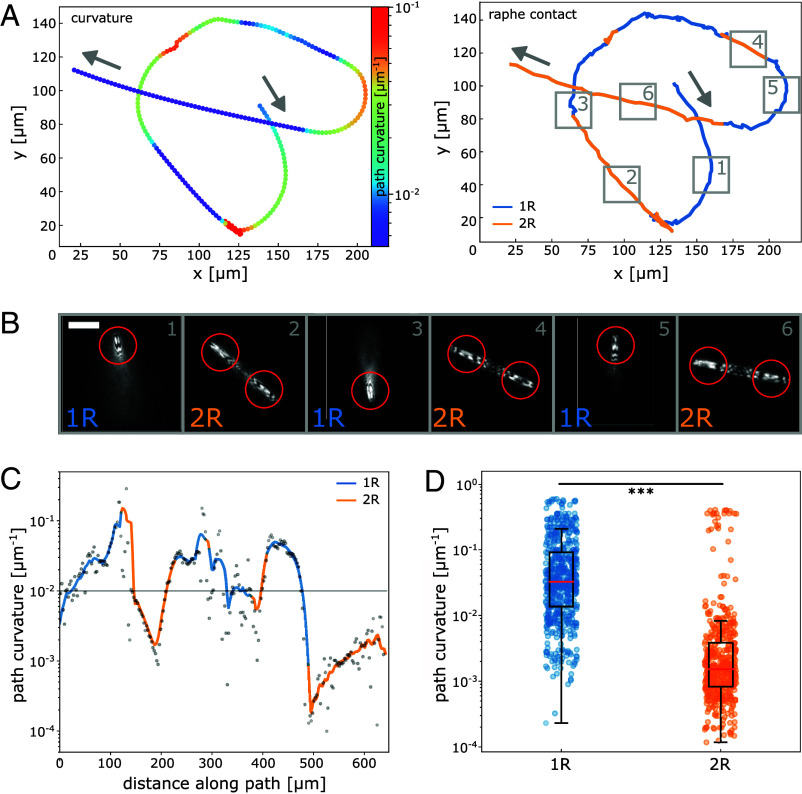
Path curvature in one-raphe and two-raphe branch contact gliding using interference reflection microscopy (IRM). (*A*) (*Left*) Trajectory of a single *C. australis* cell showing substantial changes in path curvature (color coded). Gray arrows indicate direction of travel. (*Right*) The same trajectory color-coded by the number of raphe branches in contact with the substrate during gliding (one-raphe branch contact gliding in blue, two-raphe branch contact gliding in orange). (*B*) IRM images from regions of interest along the trajectory in *A*, showing the switching between one-raphe and two-raphe branch contact gliding. Red circles indicate tracked raphes used to discriminate between one- and two-raphe branch contact gliding. The tracking data of the trailing raphe branch were used to generate the trajectories in A and B as it was in the present example in constant contact with the substrate. (Scale bar, 20 µm.) (C) Path curvature over distance along the path of the single-cell trajectory in *A*, with segments of one-raphe branch contact gliding (blue) and two-raphe branch contact gliding (orange). (*D*) Quantification of path curvatures from six different single-cell trajectories displaying significant differences in path curvature (Mann–Whitney *U* test, *P* < 0.001) between one-raphe (1R) and two-raphe (2R) branch contact gliding (average cell size of 40 µm).

To directly image if gliding cells switch between one-raphe branch and two-raphe branch contact gliding and how this would affect path curvature, we employed IRM. IRM enables detecting cell–substrate contacts while simultaneously tracking cell motility. In brief, IRM uses optical interference between light reflected from the coverslip and the cell wall. The intensity of the obtained signal is sensitive to the distance between the cell and the coverslip surface and thus can be used as a proxy for cell–substrate contact. This method allowed us to directly track the position of the cell, analyze the curvature of its trajectory and correlate this with the number of raphe segments in contact with the substrate. Fig. 4*A* depicts a trajectory from a single cell, with average path curvature values fluctuating over three orders of magnitude (Fig. 4*C* and Movie S6). Analyzing this and similar trajectories (*SI Appendix*, Fig. S7 and Movie S7), we found, that *C. australis* cells occasionally lost and reestablished contact with the substrate with one of its two raphe branches and hence switched between one-raphe and two-raphe branch contact gliding. A representative example is shown in Fig. 4*B*, where in panel 1 only one raphe branch is in contact with the surface (black and white striated signal in red circle, indicating 1R contact) and in panel 2 both raphe branches are in contact with the surface (black and white striated signal in red circles, indicating 2R contact). The resulting trajectories showed substantial increases in path curvature, when contact was lost in one raphe branch, and substantial reductions in path curvature once substrate contact with both raphes branches was reestablished (Fig. 4*C*). Analysis of six IRM trajectories that displayed switching between one- and two-raphe branch contact gliding revealed that the path curvature during one-raphe branch contact gliding was an order of magnitude higher (median of 0.033 µm^−1^) than during two-raphe branch contact gliding (median of 0.002 µm^−1^) (Fig. 4*D*). From this, we conclude that the substantial changes in path curvature indeed originate from switches between one-raphe and two-raphe branch contact gliding. Even though we can not discriminate between 2R1 and 2R2 gliding in our experimental IRM data (Fig. 4*D*) and do not know the frequencies by which the 2R1 and 2R2 modes predicted by the mathematical model (Fig. 3*E*) contribute to overall path curvature, we do note a good qualitative agreement between these two datasets (*SI Appendix*, Fig. S9). Interestingly, in our experimental data we observed that during switches from two-raphe to one-raphe branch contact gliding, traction can be lost in either the leading or the trailing raphe (Movies S6–S8). This suggests that the mechanism is independent of the direction of travel. In addition, switches from one-raphe to two-raphe branch contact gliding are often accompanied by directional reversals of cell movement, most likely because the force balance between the two raphe branches is suddenly disrupted and a tug-of-war mechanism between the competing raphe branches establishes a new direction of travel (37). Finally, we observed in our raw IRM tracking data (Fig. 4 *A*, *Right* panel) that the 1R path segments display a higher positional noise of the tracked contact points in x and y, as compared to the 2R path segments. These high frequency spatial fluctuations during 1R gliding highlight that a single contact site is likely more susceptible to random perturbations (e.g. hydrodynamic drag, severing of EPS strands) than two contact sites allowing a higher directional stability during 2R gliding. This increased level of directional flexibility at the expense of robustness during 1R gliding, translates into the higher spread of the path curvature data (Fig. 4*D* and *SI Appendix*, Fig. S8).

### Path Curvature Is Cell Size-Dependent.

**Fig. 5.**
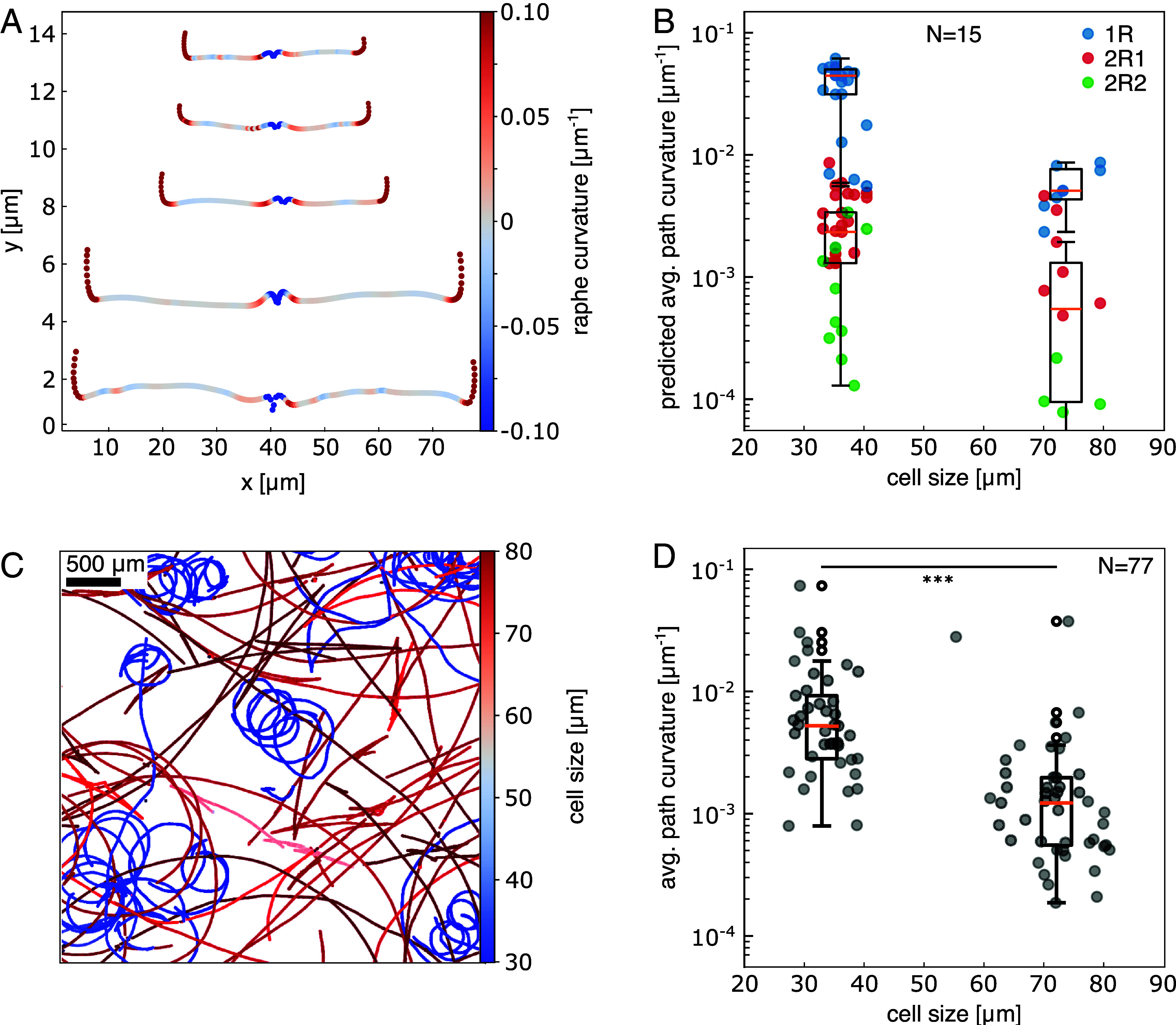
Dependence of raphe and path curvature on cell size. (*A*) Examples of 5 segmented raphes from SEM images of a *C. australis* cell population with a bimodal size distribution. Raphes are color-coded for curvature with the *Top* three from cells of size ~36 µm and the *Bottom* two from cells of size ~78 µm. (*B*) Path curvatures predicted by the mathematical model based on measured raphe geometry (average values per cell) for the different modes of gliding, from the same cell population with bimodal cell-size distribution as in *A* (average cell sizes 36.1 µm and 76.5 µm, N = 15 cells). (*C*) Trajectories of *C. australis* cells from a population with a similar bimodal size distribution as in *A*. Cells were tracked over one hour (acquired at 1 fps) in a single field of view (3.2 mm × 3.2 mm). Trajectories are color coded by the size of the respective cell. (*D*) Experimentally observed average path curvatures for each path in *C*, sorted by cell size (average cell sizes of 32.5 µm and 73.2 µm, N = 77 cells), showing a significant difference between the average path curvatures for small and large cells (Mann–Whitney *U* test, *P* < 0.001).

To validate if changes in raphe morphology led to alterations in the diatoms’ path curvature, we made use of the fact that *C. australis* undergoes asexual reproduction, resulting in progressively smaller cell sizes with each round of cell division (30). Analyzing the raphes in SEM images of 15 cell walls from monoclonal cell cultures with bimodal size distribution (average cell size of 36.1 µm and 73.5 µm) revealed that, while the general shape of the raphes remained conserved, the raphes of smaller cells exhibited more bending per unit length than the raphes of larger cells (Fig. 5*A*). Using our mathematical model to predict path curvature from local raphe geometry, we accordingly found that halving the cell size led to a five- to eightfold increase in the median predicted path curvatures in the one-raphe and two-raphe branch contact gliding modes (from 0.005 µm^−1^ to 0.044 µm^−1^ for 1R and 0.0005 µm^−1^ to 0.0023 µm^−1^ for 2R1 and 2R2, Fig. 5*B*). This cell size-dependence of the predicted path curvature was confirmed by the measured path curvature dataset of a population of cells with a similar bimodal size distribution, where a twofold reduction in cell size led to an about fivefold increase in average path curvature (Fig. 5 *C* and *D*, median path curvatures of 0.001 µm^−1^ and 0.005 µm^−1^ for large and small cells, respectively). Interestingly, during asexual reproduction, cells get shorter while maintaining their overall width. Consequently, halving the cell size reduces the cell aspect ratio (long axis to short axis) by a factor of about two. To investigate if this scaling relationship could explain the observed increase in path curvature for smaller cells, we rescaled the raphe data from a 72 µm long cell by a factor of 0.5 in the x-direction (length) to mimic a halving of the cell size. Applying our model to the rescaled raphe dataset, we find that this operation indeed significantly increases the predicted path curvatures by up to a factor of ~5 (from 0.007 µm^−1^ to 0.034 µm^−1^ for 1R, 0.0025 µm^−1^ to 0.0058 µm^−1^ for 2R1 and 0.00047 µm^−1^ to 0.0016 µm^−1^ for 2R2, *SI Appendix*, Fig. S10). Importantly, our IRM data show that the transitions between one-raphe and two-raphe branch contact gliding, along with its impact on path curvature, remained consistent regardless of cell size (*SI Appendix*, Fig. S8). Taken together, these findings demonstrate that the path curvature of *C. australis* trajectories can be predicted by the local curvature of the raphes and the dynamic switching between one-raphe and two-raphe branch contact gliding, even across a wide range of cell sizes.

## Discussion

In this study, we explored how motile diatom *C. australis* cells are able to dynamically modulate the curvature of their trajectories. While numerous studies have quantified characteristics of diatom motility through single-cell tracking and statistical analysis (5, 6, 36, 40, 41), a mechanistic understanding of how diatoms are able to dynamically modify their path shape has remained largely unexplored. Recently, Bondoc-Naumovitz at al. investigated the diversity of diatom motility and demonstrated that variations in raphe morphology across five different species are directly reflected in their trajectories (28). Nevertheless, that study did not explore the mechanisms by which diatoms dynamically adjust their trajectories during locomotion. Our experimental results and mathematical model show that *C. australis* uses two distinct modes to tune path curvature. i) Moderate changes result from shifting the regions where traction is generated along a single raphe system, supporting the earlier hypothesis that local raphe curvature at the contact site determines path shape (33). ii) Large, abrupt changes result from switching between one-raphe and two-raphe branch contact gliding. It is likely that this dual mechanism provides *C. australis* with directional flexibility across a wide range of temporal and spatial scales, without requiring cell deformation or external appendages.

Due to these mechanisms, *C. australis* cells of similar size and conserved raphe geometry, as well as individual cells at different points in time, can exhibit strikingly different trajectories. This dynamic variability underscores a key feature of diatom gliding: the ability to flexibly modulate movement despite rigid structural constraints. Our model provides a mechanistic explanation for this variability by linking the mode of cell–substrate contact and local raphe geometry. On the one hand, large radius trajectories—much greater than the cell length—originate from two-raphe branch contact gliding where both raphe branches or the rather straight central part of one raphe branch contribute to force generation (2R2, 2R1). On the other hand, tight turns occur during one-raphe branch contact gliding (1R), where the highly curved parts of one raphe branch determine the path curvature. Sigmoid paths likely result from actively shifting between raphe segments with opposing curvatures, creating alternating turns (Movie S9). Interestingly, upon directional reversals, the orientation of curvature is mostly conserved. This may reflect either i) the reversal of the intracellular force-generating machinery along a fixed raphe segment (similar to a car going back and forward with a fixed steering wheel) or ii) the conservation of orientation and magnitude of path curvature due to the bilateral symmetry of the raphe system, when the active zone switches from one raphe branch to the other. Both behaviors have previously been reported (33, 38) and are observed in our IRM experiments (Movie S8). Taken together, our findings demonstrate that the spectrum of path shapes can be linked to the local geometry of the raphe, enabling the path curvature to remain largely consistent over distances far exceeding the cell size—even during directional reversals. This contrasts with alternative theories, such as hydrodynamic propulsion models, which can neither account for the long-range preservation of path shape nor the high speed of directional reversals (8, 42).

In our study, we propose that force generation along the raphe system is spatially regulated, although the underlying mechanisms remain unclear. Recent studies demonstrated that three raphid diatom-specific myosins exhibit coordinated movement during “smooth-sustained” gliding, and opposing movements during pauses or very slow cell movement—patterns consistent with a role of these myosins in force production for motility (37). It is thus conceivable that cells spatially modulate force generation by redistributing these raphe-associated myosin motors or by locally regulating adhesive EPS secretion. As described here, *C. australis* cells exhibit abrupt changes in path curvature by lifting and detaching one side of their raphe system from the substrate, a behavior also observed in other diatom species (25, 33, 38) and reminiscent of “waving” of apicomplexa (43, 44). This lifting could result from either halting adhesive EPS secretion on one side or from force imbalances that sever adhesive EPS strands on one half of the cell. Single-cell force measurements have shown that motile diatoms can produce forces of up to 800 pN (45), sufficient to overcome the forces needed to break the EPS strands (11, 46). However, the precise location(s) where traction-force is generated may not coincide exactly with the raphe–substrate contact sites, due to the length and elastic properties of the EPS strands (11, 12, 47). Their spatial arrangement and mechanical characteristics likely influence diatom motility patterns, contribute to the characteristic jerky movements (46), and facilitate cooperative behaviors in motility (45).

Our single-cell tracking data revealed that path curvature was frequently conserved over distances greater than the cell length, and often remained unchanged even upon reversal of the gliding direction. This consistency suggests that transient fluctuations in traction force location or strength (i.e. due to variations in EPS secretion or local adhesion conditions) do not greatly influence the shape of a diatoms’ path over long timescales. As a result, we believe that the dominant contribution to overall path curvature can be attributed to more stable structural features, in particular the geometry of the raphe. Over short timescales, we sometimes observed jerky cell movements and transient stalling, which may result from opposing motor forces during periods of 2R2 gliding, e.g. tug-of-war phases (37) or snapping of the EPS strands (46). Future investigations combining traction force microscopy with fluorescent labeling of EPS strands could clarify how active force generation (on one or two raphe branches) and passive adhesion interact to govern cell motility in general, and transitions between one- and two-raphe branch contact gliding modes in particular. Obtaining more physical parameters from such experiments may also allow to extend our model to account for the directionality and location of the active and passive forces.

The ability of diatoms to dynamically adjust their path shape is crucial for their survival, as it allows the cell to follow gradients of light and nutrients as well as to avoid desiccation (20, 21, 23, 26). In particular their phototactic response to varying light intensities and wavelengths allows diatoms to actively explore their light environment and to avoid damaging high-intensity light through directed motility (19, 32). Quantitative analysis of motility patterns in different diatom species suggest that diatoms move in circular random-walk fashion, optimized for biofilm formation and nutrient foraging (36, 48). With regard to efficiently exploring complex habitats, our data reveal that on short timescales, *C. australis* cells exhibit i) short-time ballistic runs (timescale < 100 s), ii) a long-time diffusive behavior (timescale between 100 s and 1,000 s), and iii) an even subdiffusive behavior due to the nature of their circular runs (timescale >1,000 s, *SI Appendix*, Fig. S11). This behavior, similar to the run-and-tumble mechanism of bacteria (49) and the helical motion patterns of Plasmodium sporozoites (4, 50), is in line with previous work on diatoms, indicating that diatom motility under isotropic conditions may be optimized for foraging and biofilm formation (36, 46). Circular or helical paths have also been observed during chemotaxis of other fast moving cells, for example sea urchin sperm, allowing for an effective sensing length-scale much larger than the cells’ size (51). Periodic modulation of path curvature is an important strategy for these motile single-celled organisms to measure the chemo-attractant concentration at different spatial positions (52). It is thus conceivable that diatoms potentially employ a similar strategy for nutrient sensing by dynamically modulating their path curvature to robustly follow chemical gradients. While we have demonstrated that *C. australis* modulates its path under isotropic conditions, it remains to be investigated how diatoms adapt their motility in response to anisotropic environments. Recent findings of calcium-permeable ion channels (EukCats) and calcium transients during directional reversals (53, 54) suggest that calcium signaling may play a role. Elucidating how environmental cues translate into intracellular force redistribution represents a key direction for future research.

Motile diatoms typically inhabit shallow waters and intertidal zones, where gliding enables them to avoid desiccation and/or being buried under sediments that are constantly deposited by waves and currents. As such, their natural substrates (i.e., mud, sand, rocks, algae) are highly complex, three-dimensional (3D) and not straight forward to reconstitute in a laboratory setting. Although 2D coverslip assays oversimplify the complexity of natural environments, they have proven effective in revealing general principles of motility, as studies mimicking natural 3D terrains (e.g., sand, ice, cryolite) show that 2D assays reliably capture key aspects of diatom dispersal (28, 38, 55, 56). Previous bead experiments suggest that all four raphe branches can function simultaneously (6) therefore transitioning to experiments in a 3D environment may reveal additional modes of coordination and flexibility not observable on flat substrates. Taken together, our experimental results and theoretical model show that diatoms achieve directional flexibility through dynamic switching of their substrate contact sites, a strategy that likely facilitates their navigation in heterogeneous and changing habitats.

## Materials and Methods

### Cell Culturing.

*C. australis* (Cox, CCMP3328) was grown in artificial seawater medium EASW (57) in T25 cell culture flasks (Greiner, 690195) at 20 °C under a cool white lamp (OSRAM LUMILUX cool daylight L36W/865) at a light intensity of 40 and 60 μmol photons·m^−2^ s^−1^ (irradiance of ~3 W/m^2^) Cultures were kept under a 14/10 h light/dark cycle and subcultured every seven days to maintain the culture in a logarithmic growth phase, as described in (58).

### Microchamber Assembly.

Microchambers for microscopy of motile cell populations were assembled by attaching a 24 mm × 60 mm dichlorodimethylsilane-coated (DDS) coverslip onto an Ibidi sticky slide mount (Ibidi, Germany, product 80828) (59). The DDS coating on the coverslip rendered the glass surfaces hydrophobic (contact angle larger than 100 degree) and allowed for long uninterrupted runs of the diatoms. To test the effect of different surface chemistry on the shape of diatom trajectories, we repeated 1-hour single-cell tracking experiments on two alternative surfaces (*SI Appendix*, Fig. S2): i) “Easyclean” glass slides cleaned by sonication in Hellmanex (Helma, Germany), Ethanol, and water (surfaces lightly hydrophilic), and ii) commercially prepared “Ibidi-treat” slides (Ibidi-Treat µ-Slide 8 Well, product number 80826, surfaces highly hydrophilic). Before the introduction of diatoms, the chambers were flushed with artificial seawater medium EASW to remove any debris before adding an appropriate number of cells to the chamber that would result in about 100 cells per field of view (3.2 mm × 3.2 mm). Higher cell concentrations were avoided as these lead to frequent cell–cell encounters, which might affect the shape of their trajectories.

### Brightfield Microscopy.

*C. australis* cells were transferred from the culture flask to a freshly prepared microchamber and imaged at 22 °C using a Nikon Ti-2 microscope equipped with a Nikon Plan Fluor DL 4× 0.13 air objective and a Lumencor SOLA Light engine LED lamp at 2% intensity and additional 50% neutral density filter and a 530/11 nm transmission filter in the light path (irradiance at sample of 4.6 W/m^2^ = 20 µmol photons /m^2^s). Time-lapse videos were acquired using a pco.edge 4.2 sCMOS camera with a spatial resolution of 1.625 µm/ pixel and a temporal resolution of one frame per second at 15 ms exposure time over a time-course of one hour using the Nikon NIS-Elements software. For motility assessment, data were preprocessed in Fiji (https://imagej.net/), converting the movies into binary movies (reducing the dynamic range of the video to two-pixel values of 0 for black and 255 for white), using the inbuilt threshold detection method in ImageJ to reduce file-size. Tracking was achieved using a threshold detection method in the Fiji plugin Trackmate version 7.13.2, yielding the spatial coordinates for each single cell at every timepoint from which cell trajectories, velocities, and path curvature were calculated using a custom-written python script. Traces shorter than 300 s were excluded from the analysis, as we preferred to study the spectrum of single-cell path curvatures on timescales longer than 5 min. The single cell tracking additionally yielded the length of each cell’s long axis (hence cell size), as well as the orientation of each cell’s long axis at every point in time.

### IRM.

*C. australis* cells were transferred from the culture flask to a freshly prepared microchamber and imaged at 22 °C using a Nikon Ti-2 microscope. The Nikon Ti-2 microscope was set up for IRM by removing the upper filter and inserted a 50/50 mirror in the lower position filter cube where the dichroic mirror is located, optimizing the incident angle of the light in the Nikon Ti2-LAPP TIRF module for IRM by closing the aperture diaphragm to approximately 75% (100% is fully open) and installing a Nikon CFI Apochromat 60× 1.49 TIRF oil objective (60). As a light source we used the green 561 nm line from a LED Lumencor Spectra Light Engine (Lumencor) at an intensity of 1.5% to irradiate the sample from below in the inverted microscope (irradiance at sample of 12 W/m^2^ = 53 µmol photons/m^2^s). The resulting signal was recorded using a pco.edge 4.2 sCMOS camera at 10 ms exposure time with a 2 × 2 binning of the data to reduce file-size. To increase the limited field of view of a 60× objective, a tiling of 4 × 4 or 3 × 3 field of views was used, resulting in a temporal resolution of 4.16 s and 2.56 s per frame respectively. Time-lapse videos were recorded over 30 min and up to 60 min and later stitched together using the Fiji grid collection stitching plugin with a 15% overlap between the tiles. The position of substrate contacts for each cell were obtained using the Fiji plugin Trackmate v7.13.2. The inbuilt Difference of Gaussian detection mode allowed us to track the position of each contact individually and reconstitute the cells trajectory from the part of the raphe that was in constant contact with the substrate (i.e. after switching from 1R to 2R gliding, the position of the earlier contact was continued to be tracked). From the obtained x–y-data, the diatoms trajectory, path curvature, and the number of contacts in each frame were extracted using a custom-written python script. The number of contact sites were then used to color-code the cells’ trajectory to and further discriminate path curvatures between one- and two-raphe branch contact gliding.

### Assessing Path Curvature, Cell Velocity, and Offset Angle in Diatom Trajectories.

From the x-y-t datasets obtained from our single cell tracking, cell trajectories could be reconstituted and motility parameters extracted for each cell and point in time, using a custom-written python script.

To quantify the path curvature *c,* the raw x–y datasets were first preprocessed to smoothen the trajectories using a spline interpolation function. Next, the interpolated datapoints were equally spaced, conserving the total number of datapoints. Path curvature *c* was then computed by analyzing the rate of change of the tangent component of the velocity vector as described in (61):

[1] $$c=\frac{\left|\left(\frac{d^{2}x}{{\mathrm{dt}}^{2}}\cdot \frac{\mathrm{dy}}{\mathrm{dt}}-\frac{d^{2}y}{{\mathrm{dt}}^{2}}\cdot \frac{\mathrm{dx}}{\mathrm{dt}}\right)\right|}{{\left({\left(\frac{\mathrm{dx}}{\mathrm{dt}}\right)}^{2}+{\left(\frac{\mathrm{dy}}{\mathrm{dt}}\right)}^{2}\right)}^{3/2}}.$$

Cell velocity along the path *v* was obtained by first calculating the displacement in the interpolated x and y datasets between two subsequent timepoints and then taking their geometric average divided by the temporal difference between the timepoints:

[2] $$v=\frac{\sqrt{\Delta x^{2}+\Delta y^{2}}}{\Delta t}.$$

The cell offset angle α was obtained by calculating the absolute difference of the orientation of the cell’s long axis and the direction of travel φ φ. The orientation of each cell’s long axis for each point in time was obtained from our tracking data as it is a standard return for the threshold detection method in Trackmate (62). The direction of travel was then computed as follows:

[3] $$\varphi ={\text{tan}}^{-1}\left(\frac{\frac{\mathrm{dy}}{\mathrm{dt}}}{\frac{\mathrm{dx}}{\mathrm{dt}}}\right).$$

As the time resolution in our long-term single cell tracking data was limited to 1 fps, path curvatures, cell velocities, and offset angles were averaged over 4 µm along the path, using a rolling averaging function. The size of this averaging window was chosen based on the average size of the contact site in our IRM data and maintained for the analysis of raphe curvature, to ensure a similar resolution. The robustness of the described curvature analysis with regard to data binning was confirmed (*SI Appendix*, Fig. S12).

### SEM Imaging of Diatom Cell Walls.

To isolate diatom cell walls, 200 mL of *C. australis* cell culture (that was used to perform the experiments for data shown in Fig. 2) were grown to a density of ∼10^5^ cells mL^−1^. Cells were then harvested using a centrifugation of 5 min at 2,000×*g* using a Beckman-Coulter Heraeus Biofuge Stratos centrifuge. The pellet was resuspended in 10 mL extraction buffer (2% SDS, 100 mM EDTA pH 8) and continuously shaken at 55° for 1 h to solubilize intracellular material. Diatom cell walls were then washed three times by pelleting using a 5 min centrifugation step at 2,000×*g* and resuspending in with 2 mL 10 mM EDTA pH 8. Finally, the cleaned cell walls were washed in acetone, followed by centrifugation and resuspension in water. The suspension was then transitioned to 100% ethanol through a series of centrifugation-resuspension steps, with incremental ethanol concentration increases (20%, 40%, 60%, 80%, and 100%). To prevent the collapse of cell walls and preserve their 3D morphology, water-free diatom cell walls were then critical-point dried (using a Leica CPD 300) and mounted onto resin pads for sputter coating with platinum using a Baltec SCD 050 instrument and argon process gas (40 mA, 40 s). SEM images of diatom cell walls were taken using a JSM 7500F field emission scanning electron microscope (Jeol) at an acceleration voltage of 5 kV at the Electron Microscopy core facility at the CMCB Technology Platform at TU Dresden.

### Analysis of the Local Raphe Curvature.

SEM images of diatom cell walls were preprocessed in Fiji to enhance contrast. The raphes were then manually traced using the segmented line tool, and the extracted x–y coordinate data were used to reconstruct raphe shapes and analyze their curvature with a custom Python script. To ensure uniform spacing of data points, we applied spline interpolation to the x–y datasets of each raphe pair, creating a new dataset of equally spaced points with 10 points per µm (*SI Appendix*, Fig. S5*A*). Curvature was then analyzed using Eq. **1** within 4 µm windows that were moved point by point (at a resolution of 10 points per µm) along the raphe. The obtained raphe curvature values were used to illustrate local raphe curvatures in Figs. 3*B* and 5*A*.

### Mathematical Model to Predict Path Curvature Based On Raphe Geometry.

To quantitatively predict path curvatures from local raphe geometries, it was necessary to determine which regions of the raphe system could establish substrate contact, either individually or simultaneously. Using SEM images of *C. australis* cell walls, we assessed which parts of the raphe system could come into proximity with the substrate. According to previous studies (28, 33, 37), only raphe regions in direct contact with the substrate contribute to motility. Studying the shape of the *C. australis* cell wall, we find that these contact areas differ between one-raphe and two-raphe branch contact gliding (*SI Appendix*, Fig. S5*B*).

In the case of one-raphe branch contact gliding (1R) only one of the two raphe branches is in touch with the substrate and produces traction, whereas the other raphe branch is lifted up, i.e. away from the substrate, as has been previously observed (25, 33, 38). This allows the highly curved terminal regions of the remaining raphe branch to establish contact with the substrate and contribute to gliding motility and dictate path curvature. Conversely, as the central raphe regions are not in contact with the flat substrate during 1R gliding, we assume that these regions do not contribute to 1R motility. Based on geometric considerations from our SEM images (*SI Appendix*, Fig. S5 *B* and *C*, blue segments), we estimated that only the terminal 30% of each raphe branch could contribute to motility during one-raphe branch contact gliding. We excluded the final 1 µm of each terminal raphe slit, as previous TEM experiments indicated that this region is filled with solid silica and therefore most likely cannot transmit forces (63).

In the case of two-raphe branch contact gliding (2R), the cell lies flat on the substrate with the central parts of both raphe branches touching the surface. In this case, the highly curved central and terminal parts of the raphe branch cannot come into contact with the flat substrate due to the shape of the cell wall. Consequently, we assume that these regions do not contribute to 2R gliding motility and were therefore excluded from further analysis. We find that only the central portion of each raphe branch contributes to motility during two-raphe branch contact gliding, accounting for approximately 50% of the total raphe length in both small and large cells (*SI Appendix*, Fig. S5 *B* and *C*, orange segments). Since we cannot distinguish, whether forces for motility originated from a single raphe branch or both raphe branches simultaneously, we made a further distinction between two-raphe branch contact with only one active raphe branch (2R1) and two-raphe branch contact with both active raphe branches (2R2).

We then constructed a mathematical model, based on a general theory for gliding that was initially motivated by apicomplexa and now allows us to compute path curvatures for all three possible gliding scenarios (1R, 2R1, 2R2) (39). In contrast to the case of apicomplexa, which have a 2D flow field on their surfaces, for diatoms the flow field is strongly restricted by the fixed 1D geometry of the raphe, leading to a reduced version of the general theory. For simplicity, we assume constant force production and friction along the length of the raphe, neglecting the mechanical properties of the EPS strands and the dynamics of the actomyosin–motility complex.

Assumptions:

1. The raphe is assumed to be a 1D feature, described by a line parameterized in 2D as **r**(s). Here, s is parameterizing the position along the whole raphe, or subsections of it that are regarded as being in active contact with the substrate. The parameterization **r**(s) is relative to a reference point **r**_ref_, i.e. the cell center. The arc length is denoted by s. We use our theory to predict the trajectories of this point.
2. We assume that the active section of the raphe is producing a constant motion of the adhesive EPS strands in the direction of the tangent $t\left(s\right)$, with speed $\overset{¯}{v}$.
3. The whole cell moves as a rigid body with a translational velocity V and angular velocity (rotating around the reference point) Ω. As we are moving in 2D, and assume the diatom is not rotating around its long axis, but only around the z-axis, Ω is a scalar. A point on the raphe at position s is moved over the substrate due to the global motion of the cell as $V+\Omega \times r\left(s\right)$ (where the 3D cross product is used by extension).
4. We assume that a local force is produced at position s due to the friction of the adhesive EPS strand motion relative to the substrate. That means the force is proportional (with a constant that we set to 1 here) to the resulting relative velocity, obtained by adding the velocity due to cell motion and the velocity of tangential motion of the motors in the raphe. If both velocities add up to zero, the adhesion point is stationary on the substrate, and no force is produced. Hence, we obtain the force density $f\left(s\right)=\bar{v}t\left(s\right)+V+\Omega \times r\left(s\right)$.

Based on this setup, we get the motion of the cell described by V and Ω, by enforcing global force balance, $\begin{array}{c} F=\int f\left(s\right)\sqrt{g}ds=\int \left(\bar{v}t\left(s\right)+V+\Omega \times r\left(s\right)\right) \end{array}$$\begin{array}{c} \sqrt{g}ds=0 \end{array}$, where $\sqrt{g}$ is the Jacobian determinant necessary if $r\left(s\right)$ is not an arc length parameterization. Because we assume a constant force per length, and not a self-organized surface flow field like in the case of the apicomplexa, this equation directly delivers a condition for V and Ω. Paired with an analogous condition for torque balance, we get three equations (as the force equation has two components, but we only have one relevant torque perpendicular to the plane) for three unknowns $V_{x}$, $V_{y}$, and Ω. The problem can be formulated as a 3 × 3 inhomogeneous linear matrix problem, where the matrix coefficients are dependent on the raphe geometry (a simple version of the geometry tensors introduced in (39)) and can be computed by numerical integration. This problem usually has a unique solution, which gives us the velocity V and rotation around the reference point Ω that satisfies global force and torque balance. As both are constants, the resulting trajectory will be a circle (with the exception of either V or Ω vanishing, yielding a point or a line, respectively). The radius of the resulting circle is given as $R=\left|V\right|/\Omega$, which corresponds to the path curvature $\kappa =1/R=\Omega /\left|V\right|$.

In both the 1R and 2R contact zones, we assumed that traction occurs along 4 µm long raphe segments. This length is based on observations in our IRM experiments and previous observations in literature suggesting that forces for motility are created only along short segments of the raphe (33).

Consequently, we subdivided the 1R and 2R contact zones into short 4 µm sections and imported the segmented raphe geometries in Mathematica 14.2. Then i) the active regions were defined as rolling 4 µm windows constraint to the different categories (1R, 2R1, 2R2), ii) the components of the linear matrix problem were numerically computed for each window and iii) subsequently the linear equation was solved for V, Ω, and κ. In case of 2R2 gliding, this calculation was performed for all possible combinations of the 4 µm contact sites on the front and hind raphes. In summary, this resulted in the distribution of predicted path curvatures shown in Figs. 3*E* and 5*B*.

## Supplementary Material

Appendix 01 (PDF)

Movie S1. Example of a tracked cell population of about 80 individual cells in one field of view, imaged at 4× magnification and 1 fps for one hour and tracked using the Fiji-Plugin Trackmate (threshold detection method). Each cell trajectory is displayed in a different color to improve contrast between individual tracks. The raw microscopy data was preprocessed by inverting contrast and creating a binary image to reduce file size.

Movie S2. Example of an individual diatom trajectory displayed in Figure 1C, imaged at 4× magnification and 1 fps for one hour and tracked with Fiji-Plugin Trackmate (threshold detection method). The raw microscopy data was preprocessed by inverting contrast and creating a binary image to reduce file size.

Movie S3. Part of an individual motile diatom trajectory used to quantify motility parameters in Figure 2A-E. The cell was imaged at 4× magnification and 1 fps and tracked with Fiji-Plugin Trackmate (threshold detection method). The raw microscopy data was preprocessed by inverting contrast and creating a binary image to reduce file size.

Movie S4. The same cell as in supplementary Movie S3, but now with the front and back of the cell tracked individually to display the offset between the two trajectories along stretches of high curvature. The raw microscopy data was preprocessed by inverting contrast and creating a binary image to reduce file size. Tracking was done using the Difference of Gaussian detector in the Fiji plugin Trackmate.

Movie S5. Part of another individual motile diatom trajectory with the front and back of the cell tracked individually to display the offset between the two trajectories along stretches of high curvature. The raw microscopy data was preprocessed using 2×2 binning, inverting contrast and creating a binary image to reduce file size. Tracking was done using the Difference of Gaussian detector in the Fiji plugin Trackmate.

Movie S6. IRM microscopy of an individual motile diatom cell used to create panels in Figure 4 *A–D*. Contact-sites between cell and substrate were tracked using the Difference of Gaussian detector in the Fiji plugin Trackmate and colored differently to enhance contrast between individual tracks. The cell was imaged at 60× magnification and 0.25 fps.

Movie S7. IRM microscopy of another individual motile diatom showing abrupt changes in path curvature in concert with switches from two to one-raphe branch contact gliding. Contact-sites between cell and substrate were tracked using the Difference of Gaussian detector in the Fiji plugin Trackmate and colored differently to enhance contrast between individual tracks. The cell was imaged at 60× magnification and 0.25 fps.

Movie S8. Another example of IRM microscopy of an individual motile *C. australis* cell with cell-substrate contact-site tracked using the Difference of Gaussian detector in the Fiji plugin Trackmate and colored differently to enhance contrast between individual tracks. Note that the both halves of the cell alternatingly detach from the substrate during repositioning of the cell body. The cell was imaged at 60× magnification and 0.25 fps.

Movie S9. Part of an individual diatom trajectory displaying rare sigmoid path shape by changing from a clockwise to a counter-clockwise curve. Note that upon directional reversal, the direction of curvature does not change (continues as counter-clockwise), which is also rare. Cell was imaged at 4× magnification and 1 fps, then tracked with Fiji-Plugin Trackmate. The raw microscopy data was preprocessed by inverting contrast and creating a binary image to reduce file size.

## Data Availability

All raw and analyzed data, as well as the custom-written analysis scripts and the Mathematica notebook for the computer simulations used to generate the figures in this research article can be found on the Zenodo repository under https://doi.org/10.5281/zenodo.17791511 (64).
