# Supplementary material for: Dynamic switching of cell–substrate contact sites allows gliding diatoms to modulate the curvature of their paths
Source: Proc Natl Acad Sci U S A. 2026 Apr 1;123(14):e2506122123. doi: 10.1073/pnas.2506122123 (PMC13056149; doi:10.1073/pnas.2506122123)
Supplement: Supplementary file 1 — Appendix 01 (PDF) [file pnas.2506122123.sapp.pdf]

PNAS

Supporting Information for

Dynamic switching of cell-substrate contact sites allows gliding diatoms to modulate the curvature of their paths

Stefan Golfier[1,2], Veikko F. Geyer[1], Leon Lettermann[3,4], Ulrich S. Schwarz[3,4*], Nicole Poulsen[1*], Stefan Diez[1,2,5*]

[1] B CUBE - Center for Molecular Bioengineering, TUD Dresden University of Technology, 01307 Dresden, Germany

[2] Cluster of Excellence Physics of Life, TUD Dresden University of Technology, 01062 Dresden, Germany

[3] Institute for Theoretical Physics, Heidelberg University, 69120 Heidelberg, Germany

[4] Bioquant-Center, Heidelberg University, 69120 Heidelberg, Germany

[5] Max Planck Institute of Molecular Cell Biology and Genetics, 01307 Dresden, Germany

*correspondence to: schwarz@thphys.uni-heidelberg.de, nicole.poulsen@tu-dresden.de, stefan.diez@tu-dresden.de

This PDF file includes:

Figures S1 to S12
Legends for Movies S1 to S9

Supplementary Figures

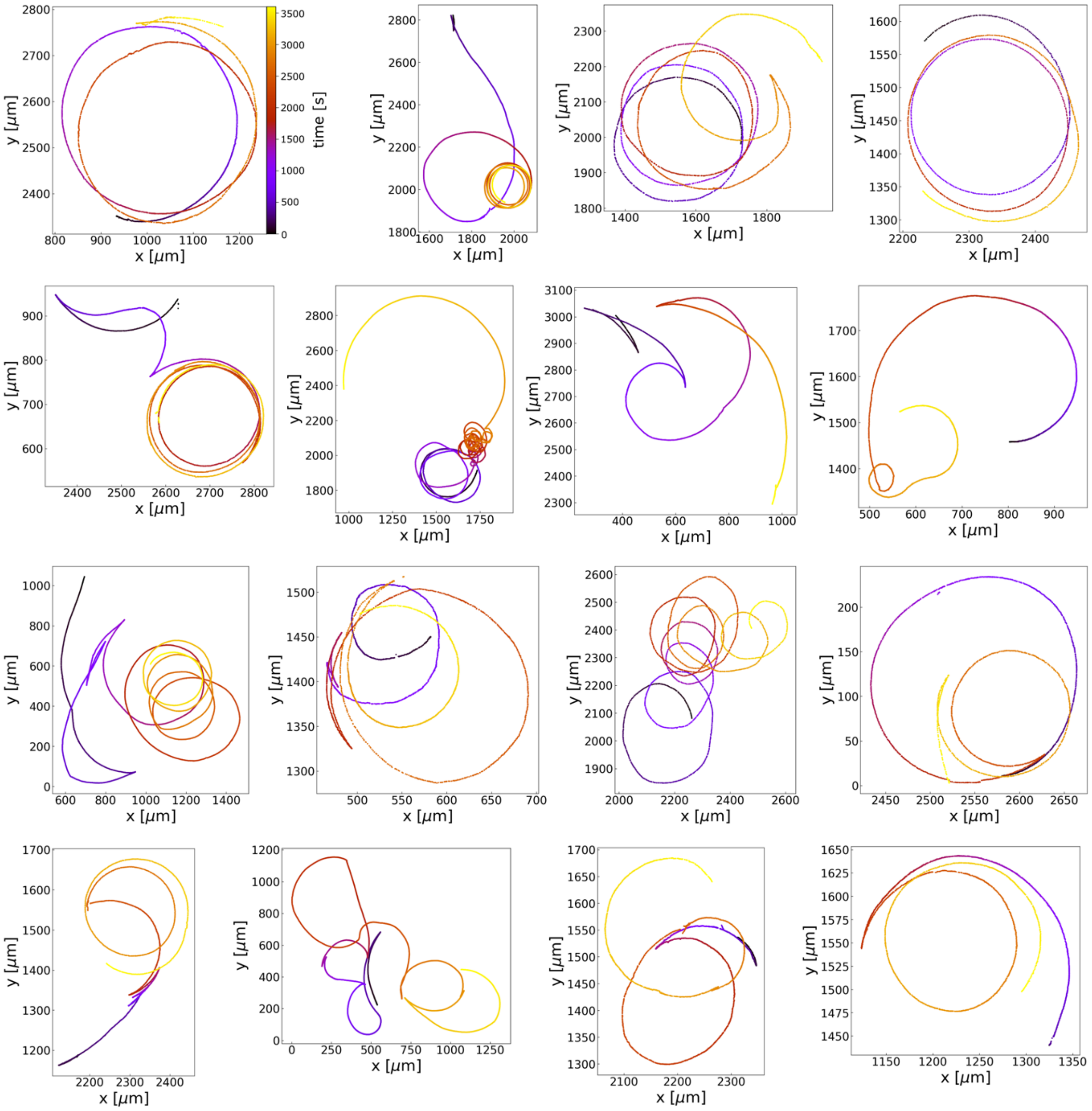

Supplementary Figure S1: Examples of single-cell trajectories of motile *C. australis*. Trajectories of individual *C. australis* cells from the population depicted in Figure 1C, tracked at 1 fps over a period of one hour (color-code is time). Cell culture with a homogeneous cell size distribution (average cell size of 25.8 µm +/- 2.0 µm).

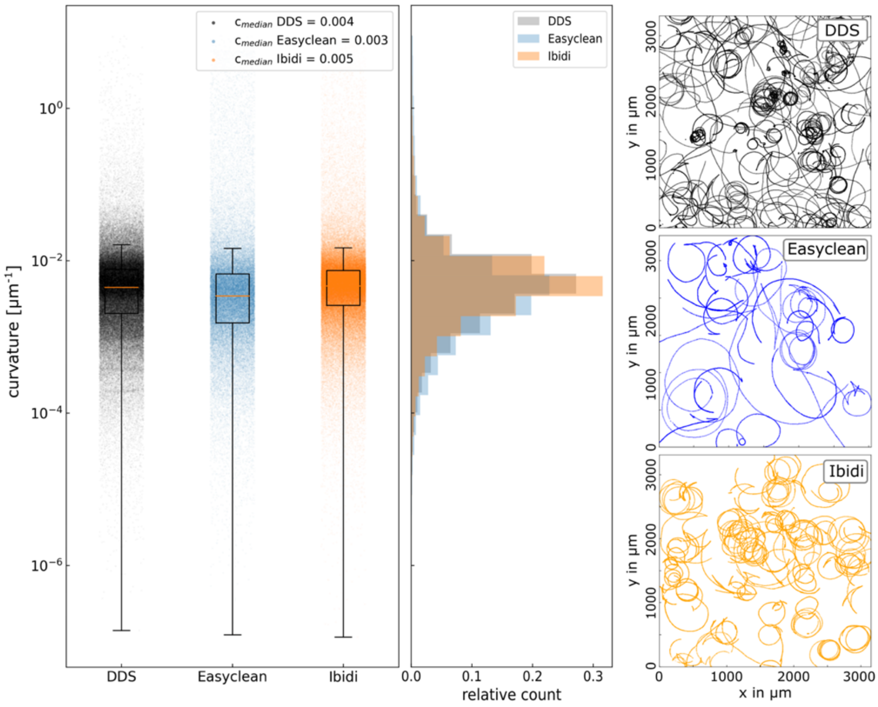

Supplementary Figure S2: Comparison of path curvatures on different glass surfaces. (Left) Spectra of path curvatures from individual cell trajectories on DDS-treated glass (black dots, 79 trajectories, same as in Figure 1C, median curvature of 0.004 μm^{-1}), 'Easyclean' glass (blue dots, 23 trajectories, median curvature of 0.003 μm^{-1}), and 'Ibidi-treat' glass (orange dots, 48 trajectories, median curvature of 0.005 μm^{-1}), averaged over 4 µm windows along the path. We found no significant difference between the three distributions (Mann–Whitney test, p = n.a.). (Middle) Histograms of the respective curvature distributions. (Right) Respective full fields of view, displaying the individual single-cell *C. australis* trajectories, acquired at 1 fps over one hour.

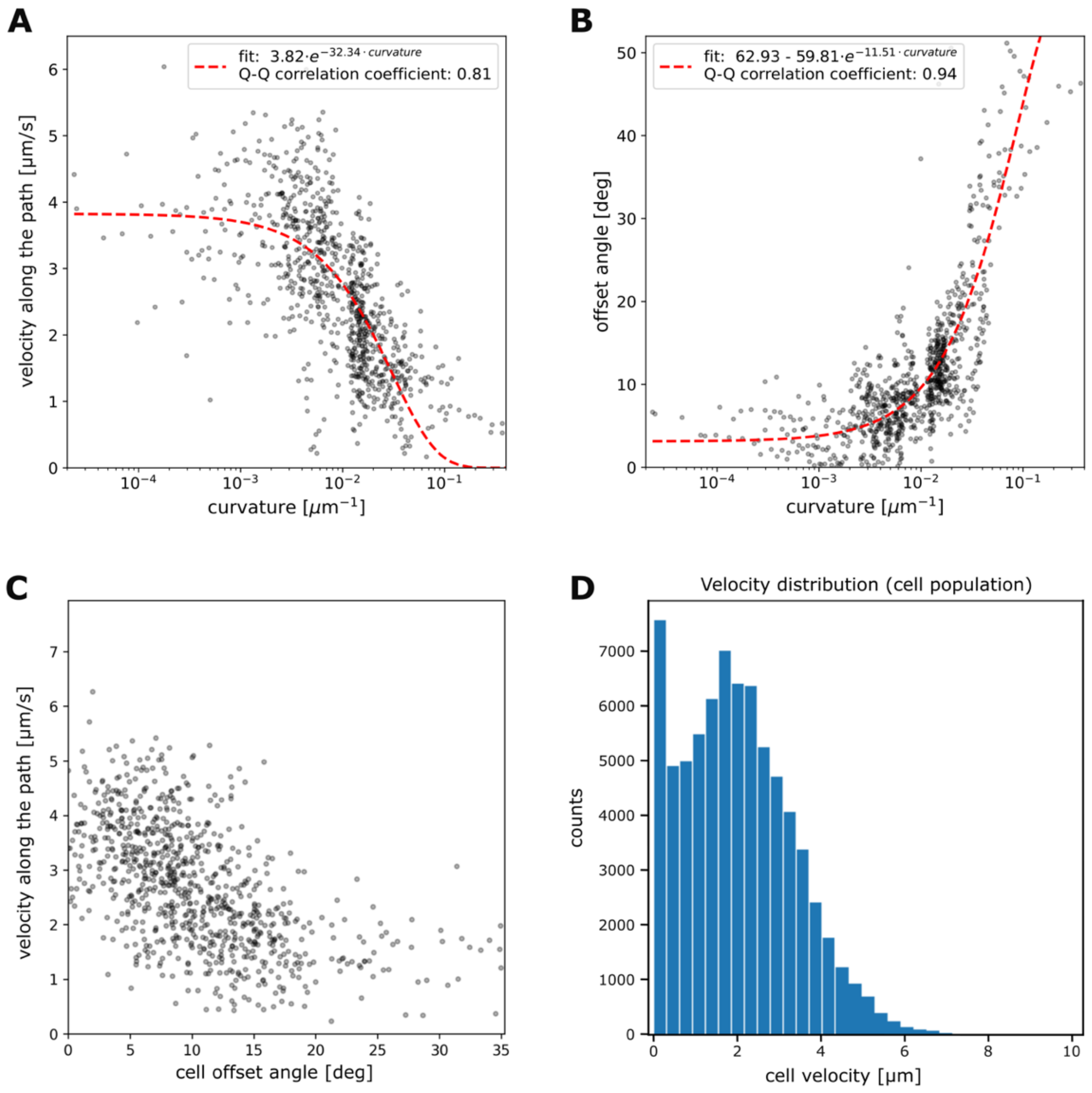

Supplementary Figure S3: Further quantification of motility parameters. A and B Cell velocity along the path (A) and offset angle (B) over path curvature for the single cell trajectory in Figure 2 A-C, with exponential fits to the data and Q-Q correlation coefficient as measure for goodness of fit. **C** Cell velocity along the path over cell offset angle for the single cell trajectory in Figure 2 A-C. **D** Histogram of all cell velocities along the path for the cell population in Figure 2 D-E.

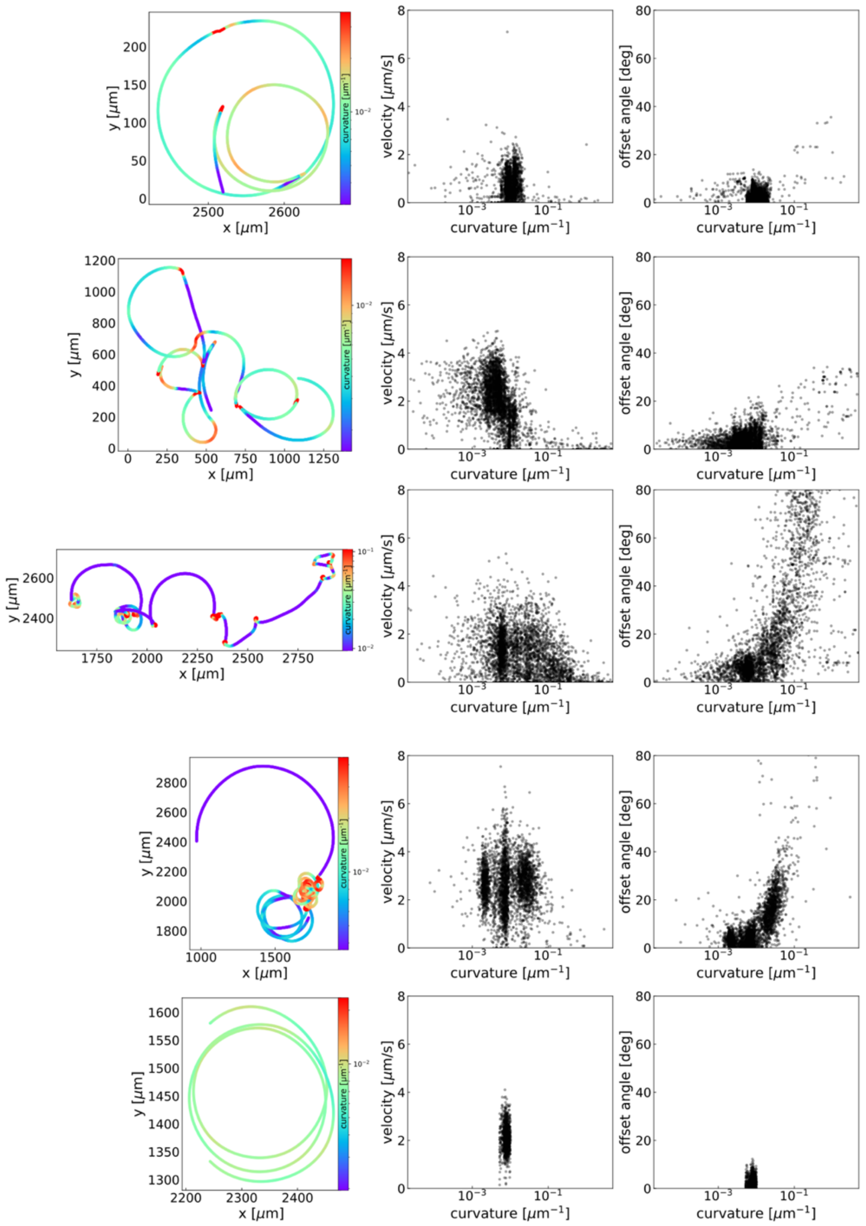

Supplementary Figure S4: Further examples of velocity - path curvature and offset angle - path curvature plots from single *C. australis* trajectories. (Left column) Trajectories of individual *C. australis* cells from the population depicted in Figure 1C, tracked at 1 fps over a period of one hour (color-code is absolute path curvature). (Middle column) Scatter plots of the distributions of the velocity along the path over path curvature of the respective single cell trajectories. (Right column) Scatter plots of the distributions of the cell offset angle over path curvature of the respective single cell trajectories. To improve clarity, axes limits in the middle and right column are the same as in Figure 2D and E.

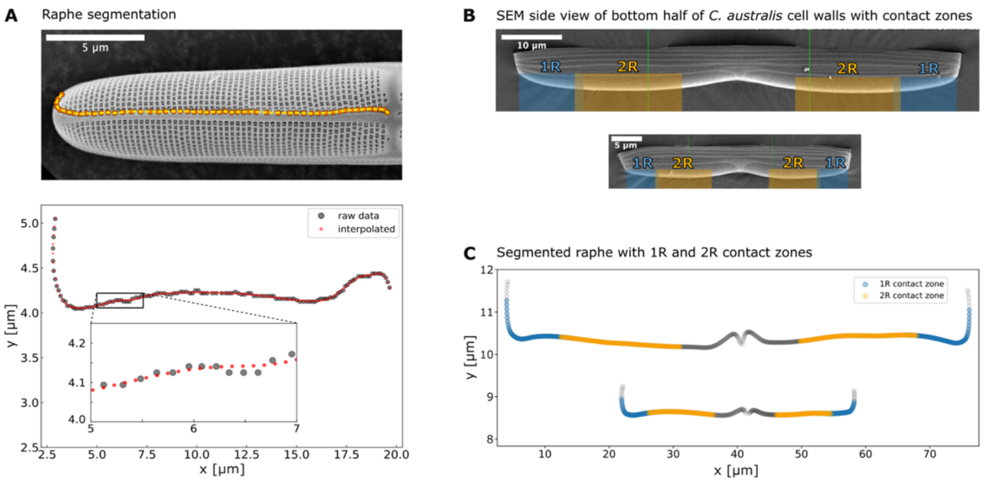

Supplementary Figure S5: Raphe segmentation and contact zone allocation. A Details on raphe segmentation and raphe curvature analysis. (top) SEM image of one half of a diatom cell wall with the manually segmented raphe branch (yellow line). (bottom) Plot of the x-y-data obtained from the segmented raphe branch with raw data in grey and interpolated data points in red. To ensure a constant spatial resolution and eliminate fluctuating distances between data points due to manual raphe segmentation, a new set of interpolated points (red dots) is set at an equal spacing with a resolution of 10 points per micrometer. **B** SEM images of the bottom halves of two *C. australis* silica cell walls from two cells of sizes ~76 µm (top) and ~38 µm (bottom) with marked contact zones for one-raphe branch (blue rectangle, terminal 30% of each raphe) and two-raphe branch (orange rectangle, center 50% of each raphe) contact gliding. Hereby, we define the contact zones based on the following: Recent studies indicate, that individual EPS strands might span the entire distance from the plasma membrane, through the raphe slit onto the substrate, reaching total extensions in the order of about 1 µm. In our SEM data of diatom frustules, we hence selected regions of the raphe that are within a 1 µm distance from a flat surface when the frustule is horizontal for 2R contact gliding, yielding stretches in the central about 50% of each raphe branch. Conversely, for 1R contact gliding, we considered the parts of the raphe branch that can come into contact with the flat substrate when the cell lifts the other raphe branch (one half of the cell body) entirely off the substrate (distance >1 µm), resulting in about 30% at the terminal part of each raphe branch. These regions naturally overlap slightly and the percentages differ between individual cells of the same size (+- about 5%) and large and small cells (+- about 10%). However, for simplicity we went with constant relative sizes of the contact zones. We confirmed the sizes of these contact zones in IRM, yielding about 32% and 47% for 1R and 2R contact gliding, respectively. **C** Segmented raphes of two cells of similar sizes as in **B**, with interpolated data points. Zones for one-raphe and two-raphe branch contact gliding are colored in blue and orange, respectively. Note that the terminal 1 µm in each raphe branch is excluded from analysis, as the terminal raphe fissure is closed and hence most likely does not allow for force transduction.

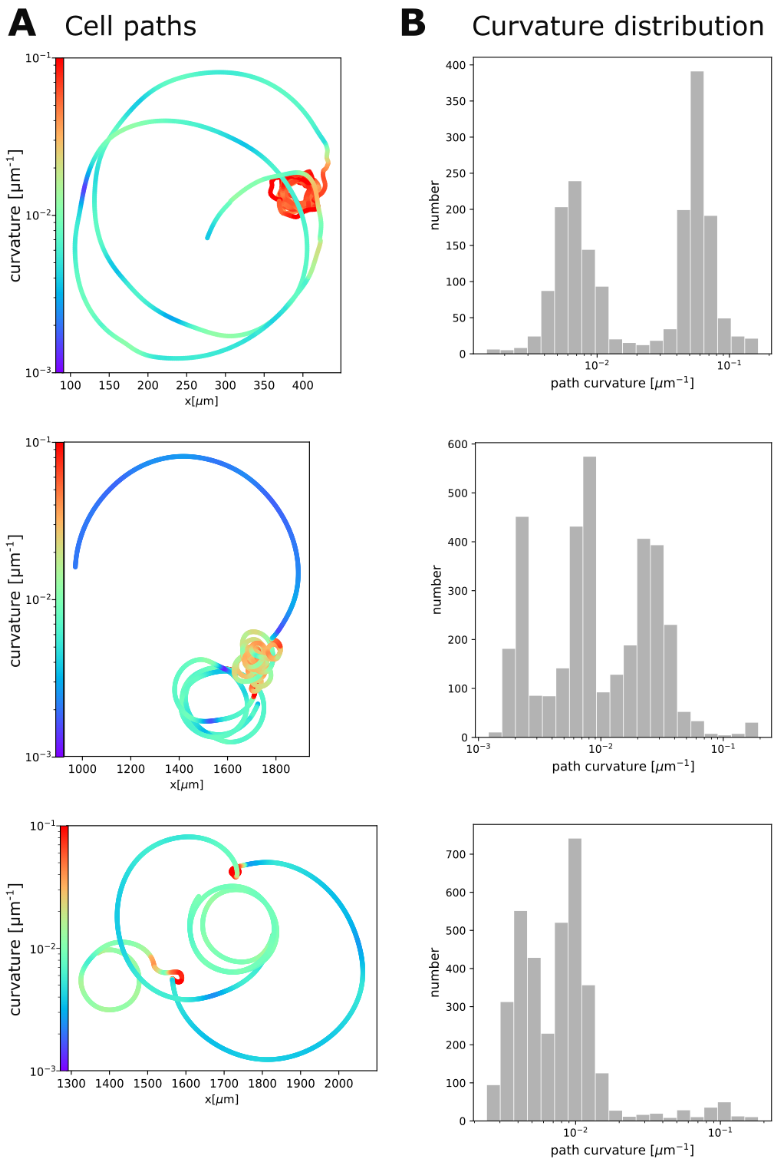

Supplementary Figure S6: Examples of multi-modal path curvature distributions indicating multiple modes of gliding motility. A Example trajectories of single *C. australis* cells of sizes 20 μm (top), 25 μm (mid) and 24 μm (bottom), tracked over one hour and color-coded for path curvature. **B** Path curvature distributions for the respective trajectories in A, revealing distinct modes of path curvatures, indicative of distinct modes of gliding motility.

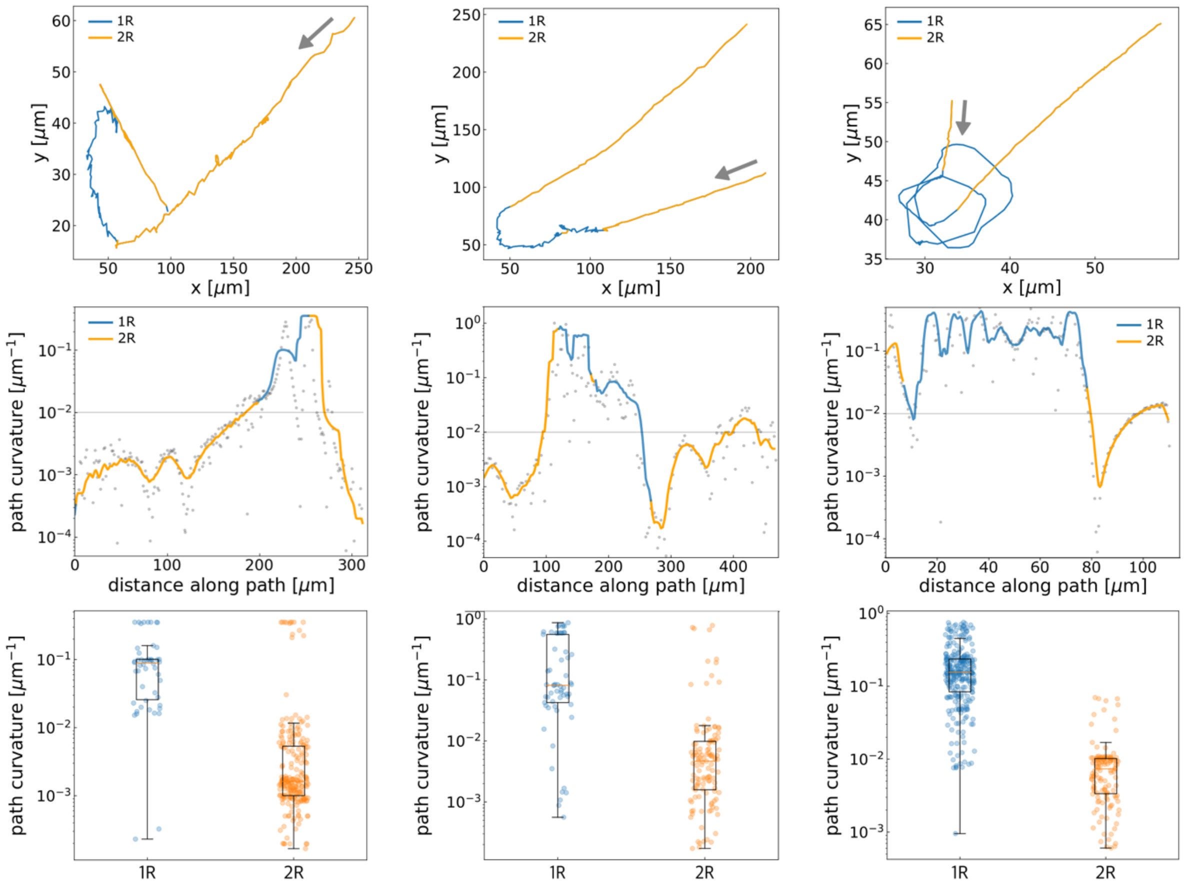

Supplementary Figure S7: Path curvature analysis during one-raphe and two-raphe branch contact gliding using interference reflection microscopy (IRM). (Top) Parts of three trajectories of single *C. australis* cells of (average cell size of 40 μm), showing abrupt changes in path curvature, color-coded for the number of raphe branches in contact with the substrate (detected using IRM) during gliding: one-raphe branch (1R) contact gliding in blue, two-raphe branch (2R) contact gliding in orange. Grey arrows indicate direction of travel. (Middle) Path curvature over distance along path of the respective single-cell trajectories with raw data as grey dots and rolling average (over window of 4 μm), color-coded for 1R contact gliding (blue) and 2R contact gliding (orange). (Bottom) Statistical quantification of path curvatures for each trajectory, pooled by 1R and 2R contact gliding (blue and orange dots respectively).

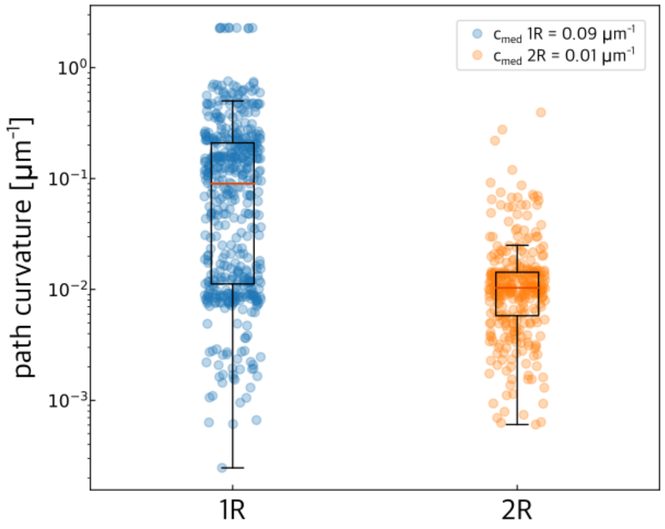

Supplementary Figure S8: Control for cell size – dependency of dynamic raphe branch switching. Quantification of path curvatures from five different single-cell trajectories showing large, abrupt changes in path curvature from cells only half the size (average cell size of 19 µm) of the cells in Figure 4 and Supplementary Figure S4 (average cell size of 40 µm). Curvature data pooled by one-raphe branch (1R) and two-raphe branch (2R) contact gliding (detected by IRM) shows, that the dynamic raphe branch switching mechanism is independent of cell size and its effect on the ensuing path curvature is conserved.

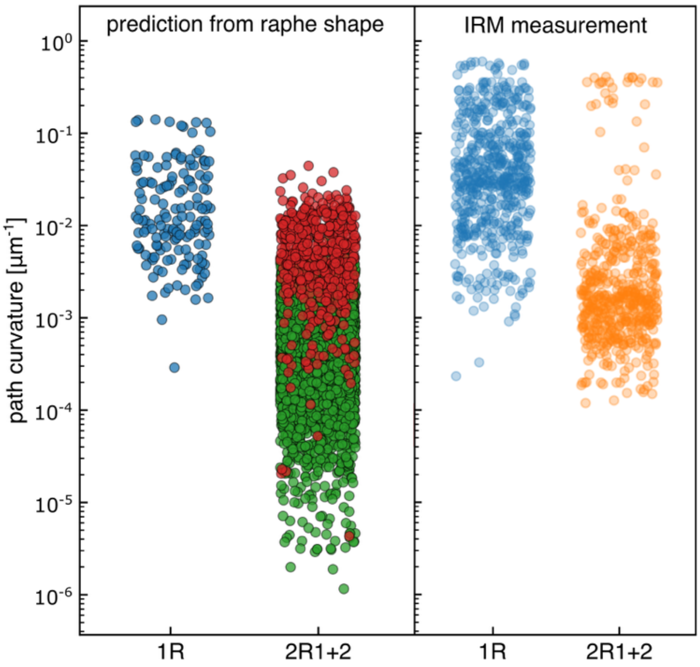

Supplementary Figure S9: Comparison of predicted and observed path curvatures across gliding modes. (Left) Path curvatures predicted by the mathematical model based on raphe geometry for each gliding mode (data from Fig. 3E; 2R1 and 2R2 pooled). (Right) Distributions of experimentally observed path curvatures for 1R and 2R gliding obtained by IRM (data from Fig. 4D). A small fraction of experimentally observed curvatures exceeds the maximum predicted by the model. In the simulations, the highest curvatures arise from the curved raphe ends, such that the curvature measured at the tracked cell center is limited to a minimum radius equal to half the cell length (~20 µm), defining the upper cut-off of the simulated data. In contrast, the highest experimental curvatures result from (i) abrupt directional reversals, which are not included in the model, and (ii) the technical limitation that in IRM we can only track the substrate-contacting raphe branch rather than the cell center, which inherently permits higher curvature values. Additionally, some high-curvature trajectories classified as 2R1+2 may originate from 1R gliding events in which the second raphe branch is already near the coverslip but not yet active, as may occur during transitions from 1R to 2R1+2.

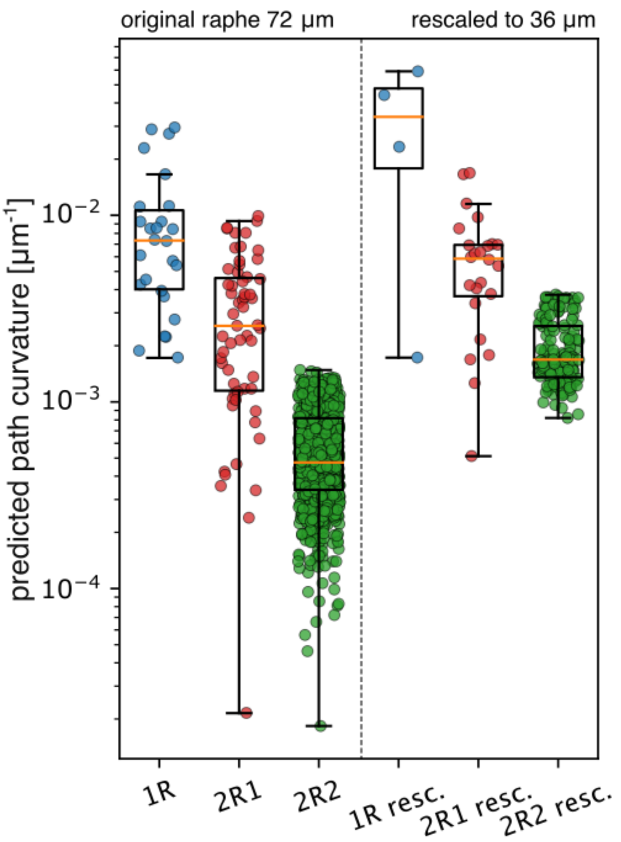

Supplementary Figure S10: Effect of raphe-scaling on predicted path curvature. (Left) Spectra of path curvatures predicted by our theoretical model, based on a SEM image of one cell of size 72 µm, for the one-raphe branch (blue dots, ‘1R’), two-raphe branch gliding contact zones (red dots, ‘2R1’) and for simultaneous activity on both raphe branches (green dots, ‘2R2’), similar to Figure 3D. (Right) The same raphe was rescaled by the experimentally observed reduction in the aspect ratio (factor of about two). Spectra of predicted path curvatures for the rescaled raphe for the one-raphe branch (blue dots, ‘1R resc.’), two-raphe branch gliding contact zones (red dots, ‘2R1 resc.’) and for simultaneous activity on both raphe branches (green dots, ‘2R2 resc.’)

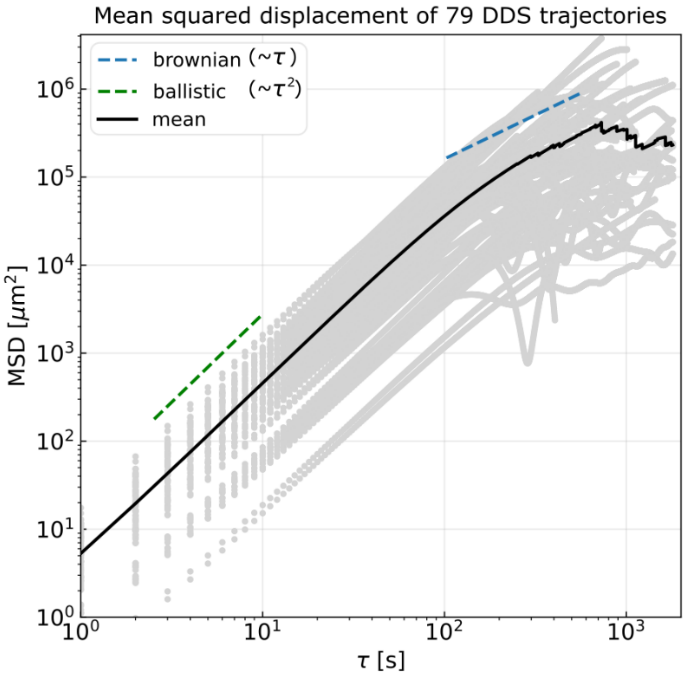

Supplementary Figure S11: Mean squared displacement analysis (MSD) of *C. australis* motility. MSD analysis of 79 single-cell trajectories of *C. australis* motility on DDS-treated glass coverslips (grey) together with the mean (black line) and dashed lines, corresponding to ballistic (green, scaling with τ^2) and diffusive (blue, scaling with τ) behaviors for reference.

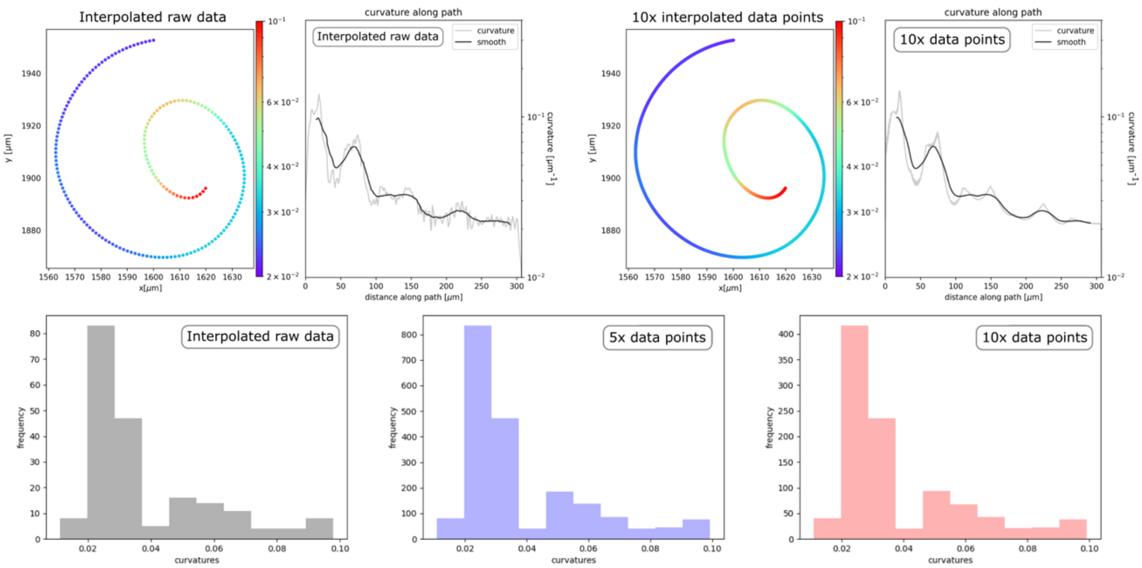

Supplementary Figure S12: Robustness of curvature analysis with regard to data interpolation. (Top left) Part of a single-cell trajectory (from Figure 2A, ~200 s) with interpolated datapoints, color-coded for absolute curvature. Corresponding curvature along the path with curvature data from interpolated points in light grey, rolling average with window size of 4 μm in black. (Top right) The same trajectory, yet with 10x the number of datapoints, generated through increasing the number of interpolated points, color coded for absolute curvature. Corresponding curvature along the path with curvature data from the interpolated points in light grey, rolling average with window size of 4 μm in black. (Bottom) Histograms of path curvature data from interpolated points for the same path segment with the original (grey), 5x and (blue) and 10x (red) number of datapoints.

Legends for movies S1 to S9

Supplementary Movie S1: Example of a tracked cell population of about 80 individual cells in one field of view, imaged at 4x magnification and 1 fps for one hour and tracked using the Fiji-Plugin Trackmate (threshold detection method). Each cell trajectory is displayed in a different color to improve contrast between individual tracks. The raw microscopy data was preprocessed by inverting contrast and creating a binary image to reduce file size.

Supplementary Movie S2: Example of an individual diatom trajectory displayed in Figure 1C, imaged at 4x magnification and 1 fps for one hour and tracked with Fiji-Plugin Trackmate (threshold detection method). The raw microscopy data was preprocessed by inverting contrast and creating a binary image to reduce file size.

Supplementary Movie S3: Part of an individual motile diatom trajectory used to quantify motility parameters in Figure 2A-E. The cell was imaged at 4x magnification and 1 fps and tracked with Fiji-Plugin Trackmate (threshold detection method). The raw microscopy data was preprocessed by inverting contrast and creating a binary image to reduce file size.

Supplementary Movie S4: The same cell as in supplementary Movie S3, but now with the front and back of the cell tracked individually to display the offset between the two trajectories along stretches of high curvature. The raw microscopy data was preprocessed by inverting contrast and creating a binary image to reduce file size. Tracking was done using the Difference of Gaussian detector in the Fiji plugin Trackmate.

Supplementary Movie S5: Part of another individual motile diatom trajectory with the front and back of the cell tracked individually to display the offset between the two trajectories along stretches of high curvature. The raw microscopy data was preprocessed using 2x2 binning, inverting contrast and creating a binary image to reduce file size. Tracking was done using the Difference of Gaussian detector in the Fiji plugin Trackmate.

Supplementary Movie S6: IRM microscopy of an individual motile diatom cell used to create panels in Figure 4 A-D. Contact-sites between cell and substrate were tracked using the Difference of Gaussian detector in the Fiji plugin Trackmate and colored differently to enhance contrast between individual tracks. The cell was imaged at 60x magnification and 0.25 fps.

Supplementary Movie S7: IRM microscopy of another individual motile diatom showing abrupt changes in path curvature in concert with switches from two to one-raphe branch contact gliding. Contact-sites between cell and substrate were tracked using the Difference of Gaussian detector in the Fiji plugin Trackmate and colored differently to enhance contrast between individual tracks. The cell was imaged at 60x magnification and 0.25 fps.

Supplementary Movie S8: Another example of IRM microscopy of an individual motile *C. australis* cell with cell-substrate contact-site tracked using the Difference of Gaussian detector in the Fiji plugin Trackmate and colored differently to enhance contrast between individual tracks. Note that the both halves of the cell alternatingly detach from the substrate during repositioning of the cell body. The cell was imaged at 60x magnification and 0.25 fps.

Supplementary Movie S9: Part of an individual diatom trajectory displaying rare sigmoid path shape by changing from a clockwise to a counter-clockwise curve. Note that upon directional reversal, the direction of curvature does not change (continues as counter-clockwise), which is also rare. Cell was imaged at 4x magnification and 1 fps, then tracked with Fiji-Plugin Trackmate. The raw microscopy data was preprocessed by inverting contrast and creating a binary image to reduce file size.
